# The JMJD family histone demethylases: structure, mechanism of action, diseases and therapeutic targets

**DOI:** 10.1186/s43556-026-00434-3

**Published:** 2026-03-20

**Authors:** Yilin Hong, Hanshi Guo, Qiang Chen, Chundong Yu

**Affiliations:** 1 Xiamen Key Laboratory of Traditional Chinese Medicine Bio-Engineering, School of Pharmacy, Xiamen Medical College, Xiamen, Fujian 361023 China; 2 State Key Laboratory of Cellular Stress Biology, Innovation Center for Cell Biology, School of Life Sciences, Xiamen University, Xiamen, Fujian 361104 China; 3 Department of Biochemistry and Molecular Biology, Zhejiang Key Laboratory of Pathophysiology, Health Science Center, Ningbo University, Ningbo, Zhejiang 315211 China; 4 Key Laboratory of Precision Medicine for Atherosclerotic Diseases of Zhejiang Province, Affiliated First Hospital of Ningbo University, Ningbo, Zhejiang 315010 China; 5 School of Life Sciences, Xiamen University, Xiamen, Fujian 361102 China

**Keywords:** JMJD family, Histone demethylases, Epigenetic, Disease pathogenesis, JMJD inhibitors

## Abstract

The Jumonji C domain-containing (JMJD) family of histone demethylases constitutes an essential class of epigenetic regulators that dynamically sculpt gene expression programs through the erasure of methyl groups from histone lysine and arginine residues. Dysregulation of these enzymes is increasingly implicated in the pathogenesis of a wide spectrum of human diseases. Yet, a fragmented, disease-specific understanding has thus far hindered a unified view of their functions across different pathological states. In this review, we provide a comprehensive and comparative analysis of the JMJD family, synthesizing their roles and mechanisms across diverse human conditions, including cancer, neurological disorders, inflammatory, autoimmune, cardiovascular, and metabolic diseases. We highlight that individual JMJD proteins can function paradoxically as both promoters and suppressors of pathology, a duality determined by the specific cellular and pathological context. A key novelty of our work is its integrated, cross-disease perspective, which moves beyond conventional silos to illuminate common pathophysiological pathways and unique regulatory networks orchestrated by these epigenetic erasers. Furthermore, we critically assess the associated therapeutic landscape, summarizing advances in the development of small-molecule JMJD inhibitors and discussing innovative strategies to tackle enduring challenges, such as enzymatic redundancy and selectivity. By integrating insights from disparate disease models, this review seeks to forge a holistic understanding of JMJD biology and accelerate the development of novel epigenetic therapeutics directed at this pivotal protein family.

## Introduction

Epigenetics, a concept first proposed by Conrad H. Waddington in 1942, refers to the regulation of gene transcription and cellular phenotype through covalent modifications of DNA or histones, chromatin remodeling, and non-coding RNAs, without altering the DNA sequences [1]. The mechanisms operate primarily through the modification of nucleosome, which comprises approximately 147 base pairs of DNA wrapped around an octamer of core histones, including H2A, H2B, H3, and H4 [2]. The N-terminal tails of these core histones protrude from the nucleosome and are subjected to a vast repertoire of post-translational modifications (PTMs), including methylation, acetylation, phosphorylation, ubiquitination, small ubiquitin-like modifier conjugation (SUMOylation), and adenylylation [3–6]. These PTMs critically influence chromatin conformation and serve as docking sites to recruit specific epigenetic regulators or transcription factors, ultimately determining gene expression patterns in response to various biological processes [7]. Among the plethora of histone PTMs, methylation on lysine and arginine residues stands out as a particularly intricate and influential code [8, 9]. The functional consequence of histone methylation is not unitary but is exquisitely dependent on the specific residue modified and its methylation state (mono-, di-, or tri-methylation). For instance, methylation of histone H3 lysine 4 (H3K4) and histone H3 lysine 36 (H3K36) is robustly linked to transcriptional activation [10], whereas methylation of histone H3 lysine 9 (H3K9) and histone H3 lysine 27 (H3K27) is frequently associated with heterochromatin formation and gene silencing [11]. For decades, histone methylation was considered a stable, irreversible mark, committing cells to long-term transcriptional programs. This paradigm was fundamentally overturned by the groundbreaking discovery of the first histone lysine-specific demethylase 1 (LSD1) [12], swiftly followed by the identification of the larger Jumonji C (JmjC) domain-containing histone demethylase family [13, 14]. These discoveries revealed that histone methylation is a dynamic and reversible process, adding a crucial layer of plasticity to epigenetic regulation.

The JMJD proteins form the largest family of histone demethylases, characterized by their conserved catalytic JmjC domain. Unlike the LSD family, JMJD enzymes are 2-oxoglutarate (2-OG, also known as α-ketoglutarate)- and Fe^2^⁺-dependent dioxygenases capable of demethylating all three methylation states (me1/2/3) on a diverse array of histone substrates, including the critical H3K9, H3K27, and H3K36 residues [15]. This enzymatic mechanism directly couples epigenetic regulation to cellular metabolism, as the reaction consumes 2-OG and generates succinate as a byproduct, rendering JMJD activity sensitive to cell metabolism [15]. While the involvement of JMJD proteins in cancer is extensively characterized, their functional significance extends far broader than oncogenesis. Emerging evidence underscores their critical roles in a broad spectrum of fundamental physiological processes, such as neurological disorders [16], inflammatory and autoimmune diseases [17], as well as cardiovascular diseases [18], and metabolic homeostasis [19]. Consequently, dysregulation of JMJD-mediated epigenetic signaling is now implicated in a growing number of human diseases across multiple pathological contexts.

However, research on JMJD has largely advanced in siloes, with its pathophysiological roles dissected predominantly within isolated disease contexts. This fragmented landscape obscures a unified understanding of their core biological principles and the remarkable context-dependent duality that defines their actions in vivo. Moving beyond their oncogenic functions [17, 19, 20], this review establishes the structural and mechanistic foundations of the JMJD family, and then decodes their complex and paradoxical mechanisms across a broad pathological spectrum, detailing both enzyme-dependent and -independent modes of action. By culminating in a discussion on the translational potential and challenges of targeting these epigenetic regulators, we aim to bridge disparate fields, illuminate cross-disciplinary insights, and chart a course for novel therapeutic strategies rooted in a fundamental, integrated understanding of JMJD biology.

## Structural features of the JMJD family

**Fig. 1:**
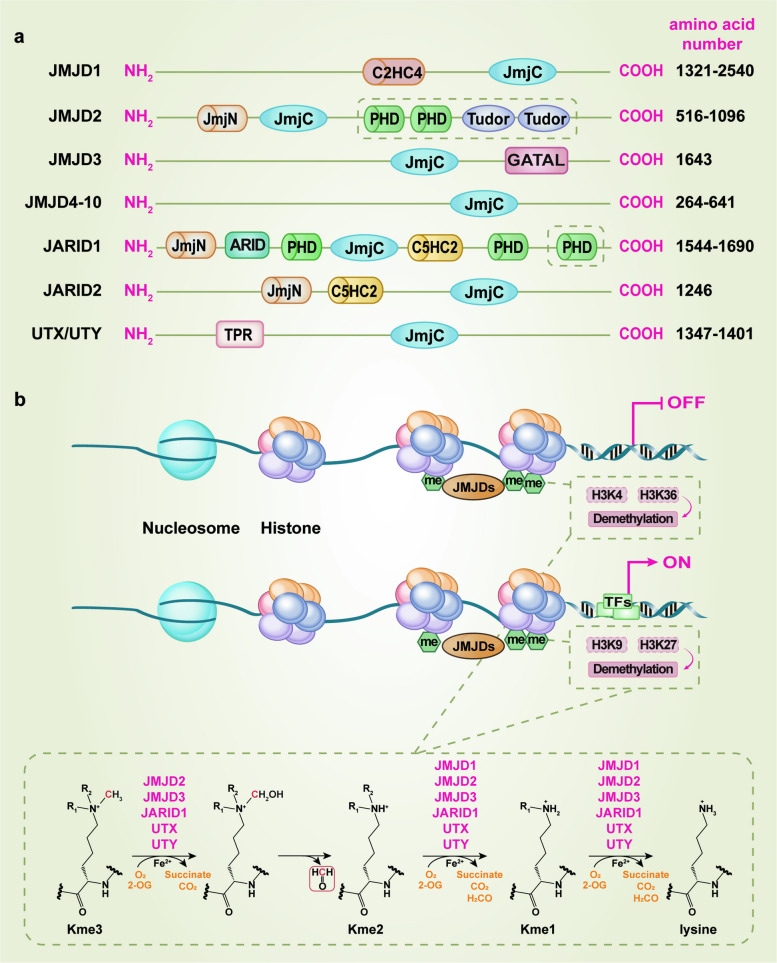
Domain architecture and catalytic mechanism of JMJD family demethylases. **a** Schematic representation of the domain structures of major JMJD subfamilies. All members share the conserved catalytic JmjC domain. The domains enclosed by dashed boxes represent different structural domains in the JMJD2 and JARID1 subfamilies. Distinct auxiliary domains (*e.g.,* Zinc fingers, Tudor, ARID, PHD, TPR) define subfamily identity and contribute to substrate specificity and protein–protein interactions. **b** The conserved dioxygenase reaction mechanism. The demethylation/hydroxylation reaction follows a classical dioxygenase mechanism involving three consecutive steps. First, the substrate (*e.g.,* methylated histone), Fe^2^⁺, 2-OG, and O₂ bind sequentially to the active site. Next, oxygen activation occurs upon O₂ binding to the Fe^2^⁺−2-OG complex, leading to heterolytic O–O bond cleavage. This step generates a highly reactive Fe^4^⁺ = O (ferryl) intermediate while decarboxylating 2-OG to succinate and CO₂. Finally, the ferryl species mediates substrate oxidation. In the case of methylated lysine, hydroxylation of the methyl group produces an unstable hydroxymethyl adduct that decomposes to release formaldehyde and yield demethylated lysine. For hydroxylation reactions, direct insertion of a hydroxyl group into an aliphatic C-H bond results in a stable hydroxylated product. Upon reaction completion, the active site is regenerated to its original state, facilitating further catalytic turnover. Abbreviations: 2-OG, 2-oxoglutarate; ARID, AT-rich interaction domain; C2HC4, Cys_2_-His-Cys_4_; GATAL, GATA-like zinc finger domain; JmjC, Jumonji C domain; JmjN, Jumonji N domain; PHD, plant homeodomain; TPR, tetratricopeptide repeat

The human JMJD protein family represents a major class of epigenetic and non-epigenetic regulators, with bioinformatic and phylogenetic analyses identifying over 30 members in humans, most of which are predicted or confirmed to function as Fe^2^⁺ and 2-OG-dependent oxygenases [21]. Based on domain architecture and sequence homology, this family comprises several core subfamilies, including JMJD1 (KDM3), JMJD2 (KDM4), JMJD3-8, JMJD10 (also known as MDIG or MINA53), JARID (KDM5), and UTX/UTY, as schematically summarized in Fig. 1a. The defining and unifying structural feature of this family is the conserved JmjC domain, which serves as the catalytic core module. This domain, approximately 170 amino acids in length, adopts a characteristic distorted double-stranded β-helix (DSBH) fold. This barrel-like DSBH structure, formed by major and minor β-sheets, creates the binding site for essential cofactors: Fe^2^⁺ is coordinated within the active site, and 2-OG binds in a bidentate manner, with its binding stabilized by electrostatic and hydrogen-bonding interactions [21–23]. While the JmjC domain provides the enzymatic core, the functional diversity and substrate specificity across the family are largely determined by a variety of auxiliary non-catalytic domains. These include plant homeodomain (PHD) fingers, zinc fingers, Tudor, AT-rich interaction domain (ARID), and tetratricopeptide repeat (TPR) domains, which are involved in binding proteins, RNA, or DNA [24]. This modular structure allows JMJD proteins to participate in complex biological processes, with their most well-characterized function being the demethylation of histone lysine residues, leading to the classification of many members as lysine demethylases (KDMs) [25, 26]. The diverse combinations of these domains not only determine the catalytic specificity of each member toward histone or non-histone substrates but also confer functional diversity, enabling their involvement in complex biological processes such as transcriptional regulation, cell differentiation, metabolism, and disease pathogenesis.

The JMJD1 subfamily (JMJD1A/B/C) is characterized by the presence of a JmjC domain and a Cys_2_-His-Cys_4_ (C2HC4) zinc finger domain [27, 28]. Among this group, JMJD1A and JMJD1B share a high amino acid sequence homology of 59.64%, while their similarity with JMJD1C is less than 50% [29], suggesting that JMJD1C may be evolutionarily more divergent. Functional studies, notably by Yamane et al., demonstrated that this zinc finger domain is crucial for enzymatic activity [30]. JMJD1A, one of the first JmjC-domain histone demethylases identified, targets H3K9me1/2 [30], while JMJD1B has also been shown to demethylate arginine marks such as H4 arginine 3 dimethylation (H4R3me2) [31]. The JMJD2 subfamily (JMJD2A-F) exhibits greater structural complexity [32, 33], among which JMJD2E and JMJD2F are considered probable pseudogenes [34]. Members like JMJD2A-C contain, in addition to the JmjC domain, a JmjN domain, two Tudor domains, and two PHD-type zinc fingers. The JmjN domain forms a composite structure with the JmjC domain to support catalysis, while the Tudor and PHD domains likely enhance stability and substrate engagement [35, 36]. JMJD2A demethylates both repressive (H3K9me2/3) and active (H3K36me2/3) marks, conferring a dual regulatory role [37]. In contrast, JMJD2D lacks the Tudor and PHD domains, resulting in a smaller protein [32].

The group encompassing JMJD3-8 and JMJD10 displays remarkable structural and functional diversity. JMJD3 contains a C-terminal JmjC domain and a GATA-like (GATAL) zinc finger domain that coordinates Zn^2^⁺ [38]. Rather than serving merely as a scaffold, this GATAL domain participates directly in DNA binding and appears to guide the locus-specific recruitment of JMJD3, thereby shaping its involvement in essential processes such as macrophage activation and cellular differentiation [39]. In contrast to chromatin-associated demethylases, several JmjC proteins, including JMJD4 and JMJD7, adopt compact architectures lacking canonical chromatin-interacting domains [40, 41]. These structural features often include dimerization elements or dedicated substrate-recognition modules, aligning with their roles as hydroxylases or multifunctional enzymes [18]. JMJD4 is a 2-OG- and Fe^2^⁺-dependent hydroxylase that is two- to threefold shorter than canonical JmjC KDMs and lacks recognizable chromatin-binding or auxiliary domains. This compact structure correlates with its exclusive hydroxylase activity on non-histone substrates, including the translation factor eukaryotic release factor 1 (eRF1) [40]. Currently, research on JMJD4 remains limited, although it has been implicated in diseases such as dilated cardiomyopathy and renal cell carcinoma, its broader physiological roles remain to be elucidated [42]. However, the functional role of JMJD5 remains a subject of debate. Some studies describe it as an H3K36me2 demethylase [43], whereas rigorous structural and biochemical studies characterize it as a substrate-specific arginyl hydroxylase, modifying proteins such as RCC1 domain containing 1 (RCCD1) and ribosomal protein S6 (RPS6) [44]. Moreover, the presence of a unique peptidase domain suggests that JMJD5 may possess the capacity to cleave at methylated arginine sites, distinguishing its mechanism from typical JmjC-mediated demethylation [45, 46]. JMJD6 represents a multifunctional enzyme capable of both lysyl hydroxylation and arginine demethylation (*e.g.,* H3R2me2, H4R3me2), making it the primary arginine-directed demethylase within the JMJD family [47, 48]. Although it lacks chromatin-binding modules, JMJD6 relies on dimerization and winged-helix structural motifs to engage its substrates. Similarly, JMJD7 is reported to share peptidase-related biochemical features with JMJD5, while also functioning as a hydroxylase for lysyl residues in GTPases [45, 46]. JMJD8 contains structural elements that facilitate its interaction with pyruvate kinase M2 (PKM2), thereby linking its function directly to cellular metabolism [49]. As a non-histone demethylase, JMJD8 targets and inhibits protein kinase B (AKT1) by removing trimethyl-lysine modifications [50, 51]. This activity contributes to its involvement in diverse pathological and physiological processes such as tumor immune evasion, adipocyte inflammation, and modulation of insulin sensitivity [50, 52, 53]. JMJD10 features a single JmjC domain with no additional functional modules [54], which enables it to catalyze protein hydroxylation of ribosomal protein L27a (Rpl27a) as well as histone demethylation of H3K9me3 [55]. Subsequent studies further expanded its substrate spectrum, revealing roles in the repression of H3K36me3 [56], H3K27me3, and H4K20me3 [57].

The JARID subfamily, primarily functioning as H3K4 demethylases, is characterized by a diverse and elaborate array of auxiliary domains that orchestrate their recruitment and regulatory specificity [58]. A representative member, JARID1A, consists of 1690 amino acids and harbors seven highly conserved domains including the JmjN domain, the ARID, the JmjC domain, the zinc finger domain, and three PHD domains. Critically, the ARID domain is indispensable for substrate recognition, as it specifically binds to cis-acting DNA elements containing “CCGCCC” motifs, thereby targeting the enzyme to specific genomic loci for the demethylation of H3K4me1/2/3 and subsequent transcriptional regulation [58, 59]. JARID1B, sharing a similar multi-domain architecture, is distinguished by the presence of an additional PLU1 motif, which may contribute to its specific functional interactions as both an H3K4 demethylase and a transcriptional repressor of tumor suppressor genes [60, 61]. Other members, JARID1C and JARID1D, possess comparable domain organizations and catalyze the demethylation of H3K4me2/3, playing crucial roles in processes such as DNA replication initiation and gene regulation [62, 63]. In contrast, JARID2 represents a unique, catalytically inactive member within the family. Although it retains the JmjN, the ARID, and a JmjC-like domain, critical amino acid mutations within the putative JmjC domain abolish its enzymatic activity [64]. Instead, JARID2 functions as a structural scaffold and transcriptional repressor. It retains nuclear localization signals and utilizes its ARID domain for DNA binding [64]. Importantly, JARID2 plays a pivotal role in chromatin complex recruitment, notably by facilitating the recruitment and activation of the polycomb repressive complex 1 (PRC1) and interacting with polycomb repressive complex 2 (PRC2), thereby participating in gene silencing through H3K27 methylation [64, 65].

UTX and UTY, encoded by the X and Y chromosomes respectively, constitute a structurally homologous pair within the JMJD family, with their shared architecture central to their function as H3K27 demethylases. Both proteins are built upon a conserved scaffold consisting of a TPR domain and a JmjC catalytic domain [66, 67]. The TPR domain is a key protein-interaction module that mediates complex formation and is required for the demethylase activity of UTX on the H3K27me1 substrate, though it is dispensable for activity against H3K27me2/3 [68]. Their catalytic cores are highly conserved, with the JmjC domains of UTX and UTY sharing up to 96% sequence similarity, which underlies their common substrate targeting of methylated H3K27 [68, 69]. Despite this high structural similarity, a critical functional divergence exists: UTX is a potent demethylase for H3K27me1/2/3, whereas UTY was initially characterized as catalytically impaired. Subsequent biochemical analysis confirmed that UTY possesses intrinsic demethylase activity against H3K27me3, yet it shows much lower activity than UTX, owing to subtle structural variations rather than domain loss [69]. This enzymatic activity enables UTX to play a crucial role in regulating developmental and disease-related gene programs by removing the repressive H3K27me3 mark. Furthermore, UTX and UTY display functional distinctions in immune regulation despite their structural homology, with each contributing differently to pro- and anti-inflammatory microglial phenotypes, which may underlie sex-specific regulatory differences [70].

Overall, the JMJD family members share a conserved JmjC catalytic core, yet they exhibit substantial diversity in auxiliary structural modules, domain complexity, and the extent of functional characterization [21]. Among them, the JMJD1 and JMJD2 subfamilies, along with JARID proteins and UTX, represent the most extensively characterized groups, featuring well-defined multi-domain architectures and firmly established roles in histone demethylation, chromatin regulation, and transcriptional regulation [27, 28, 30, 33, 58, 68]. In contrast, members such as JMJD4, JMJD7, and JMJD10 possess compact architectures lacking canonical chromatin-interacting modules and primarily function as hydroxylases, with their broader physiological significance still emerging [40, 41, 45, 55]. JMJD5 occupies an intermediate position, as its enzymatic activity remains debated and may involve hydroxylation or peptidase functions rather than conventional demethylation [43–46]. Furthermore, enzymes such as JMJD6 and JMJD8 extend the functional landscape of the family to non-histone substrates, mediating diverse biological processes including transcriptional modulation, RNA processing, cellular metabolism, and immune regulation [47, 49, 50]. Taken together, this diversity in structural organization and functional characterization highlights the JMJD family as a versatile group of epigenetic and non-epigenetic regulators, with individual members exhibiting distinct biological roles and varying degrees of research maturity.

## Biological mechanisms of JMJD demethylases

**Fig. 2:**
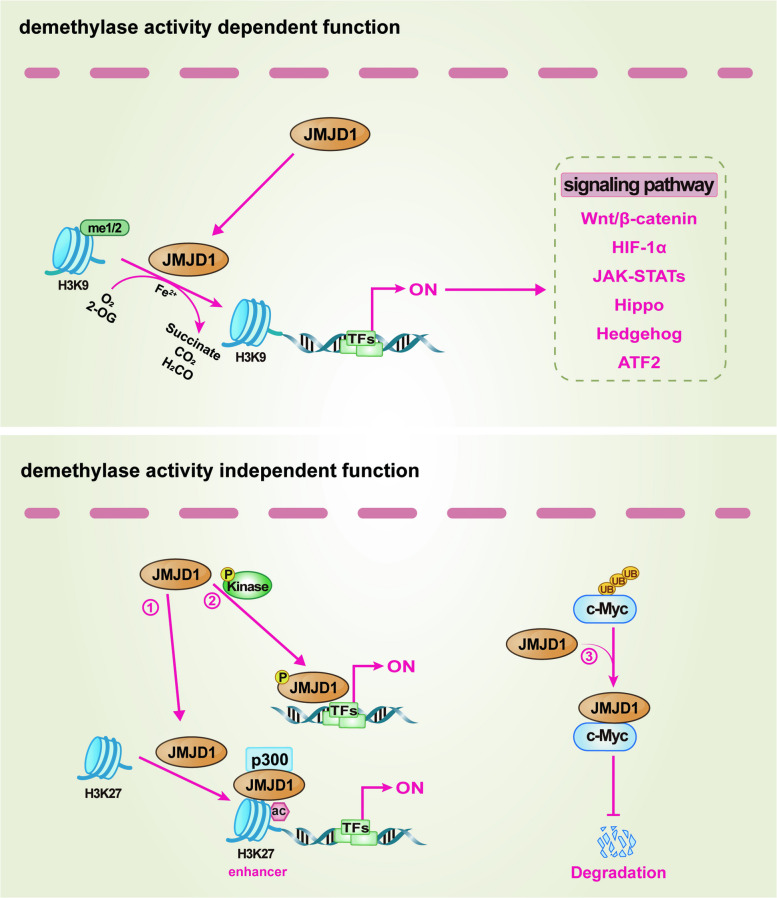
Representative diagram of JMJD family members drive pathological processes through both enzymatic and non-enzymatic mechanisms. Upper panel, Catalytic activity-dependent pathways. JMJD1 erases specific methylation marks on histones, thereby reprogramming the epigenetic landscape and gene expression, and consequently modulating multiple signaling pathways including Wnt/β-catenin, HIF-1α, JAK-STATs, Hippo, Hedgehog, ATF2, etc. Lower panel, Catalytic activity-independent pathways. JMJD1 orchestrates pathogenic gene programs via (1) the recruitment of acetyltransferases like p300, (2) phosphorylation by kinases, and (3) the stabilization of oncoproteins. Abbreviations: ac, acetylation; me, methylation; 2-OG, 2-oxoglutarate (α-ketoglutarate); ATF2, activating transcription factor 2; HIF-1α, hypoxia-inducible factor-1 alpha; JAK-STATs, Janus kinase-signal transducer and activator of transcription signaling pathway; TFs, transcription factors; UB, ubiquitination

Built upon a conserved structural framework, the JMJD family functions as a pivotal class of epigenetic erasers that dynamically sculpt the cellular epigenome and proteome, thereby translating diverse signals into precise changes in gene expression and cellular function. Central to this regulatory capacity is a Fe^2^⁺- and 2-OG-dependent dioxygenase reaction within the catalytic JmjC domain [21, 22] (Fig. 1b). Despite this shared enzymatic core, diversification in substrate recognition, regulatory domains, and cellular context has endowed JMJD members with remarkable functional specificity. This enables them to regulate a wide spectrum of biological processes via both canonical demethylase (or hydroxylase) activity and non-catalytic scaffolding functions. A representative schematic diagram was employed to delineate the molecular mechanisms by which JMJD1 proteins in driving different pathological processes such as cancer and inflammation, encompassing both their demethylase activity-dependent and -independent pathways (Fig. 2). Furthermore, their activities are further refined by upstream signals via PTMs, allowing rapid integration of diverse cellular signals. This section details the enzymatic mechanisms of the major JMJD subfamilies, providing the foundational molecular basis for understanding their roles in physiology and pathology.

### JMJD1 subfamily

The JMJD1 subfamily primarily functions to demethylate H3K9me1/2, a mark associated with transcriptional repression [27]. JMJD1A is a key physiological regulator. Its molecular function extends beyond histone demethylation to include demethylase-independent protein stabilization. For instance, it can stabilize transcription factors by blocking their ubiquitin-mediated degradation [71]. JMJD1A activity is rapidly modulated by phosphorylation; for example, protein kinase A (PKA)-mediated phosphorylation promotes its recruitment to target gene promoters in response to specific signals [72, 73]. JMJD1B also targets H3K9me2 and additionally demethylates H4R3me2, linking it to arginine methylation dynamics [31]. JMJD1C primarily functions as a transcriptional coactivator, often through non-catalytic mechanisms such as facilitating the formation of biomolecular condensates or stabilizing transcription complexes [74, 75]. The dysregulation of these core molecular functions, such as erroneous gene activation, aberrant stabilization of oncoproteins, or disrupted coactivator complexes, forms the mechanistic basis for their involvement in diseases ranging from metabolic disorders to cancer.

### JMJD2 subfamily

The JMJD2 subfamily uniquely demethylates both repressive (H3K9me2/3) and active (H3K36me2/3) histone marks. This dual enzymatic capability underpins its function as a precise and context-dependent modulator of gene expression, mechanistically enabling either the activation of silenced genes via the removal of H3K9me3 or the modulating of transcriptional elongation through the erasure of H3K36me3 [76–78]. Their enzymatic specificity varies, with JMJD2A showing a preference for trimethylated residues in vivo [37], and key residues like histidine 192 and serine 200 in JMJD2D being essential for activity [32, 79]. Furthermore, the activity and stability of JMJD2 proteins are regulated by cellular metabolites (*e.g.,* 2-OG levels) and upstream signaling, exemplified by extracellular signal-regulated kinase (ERK)-mediated phosphorylation that stabilizes JMJD2B under stress [80, 81]. This integration of intrinsic catalytic versatility with extrinsic regulatory inputs allows individual JMJD2 members to drive opposing biological outcomes, including gene activation and repression, in distinct genomic and cellular environments.

### JMJD3 and UTX/UTY subfamily

JMJD3 and UTX specifically catalyze the demethylation of the potent repressive mark H3K27me3, positioning them as critical antagonists of Polycomb-mediated gene silencing [82]. Their core molecular function is to remove this broad epigenetic barrier to permit context-dependent gene expression. JMJD3 is often inducible by environmental signals like cytokines, allowing dynamic gene activation in response to stimuli [83, 84]. This mechanism is critically involved in pathological processes, as demonstrated in a model of LPS-induced neuroinflammation and depression, where pro-inflammatory signaling through the Toll-like receptor 4 (TLR4)-nuclear factor kappa-B (NF-κB) axis induces JMJD3 to drive the expression of inflammatory genes [84]. In contrast, UTX is frequently found within stable multi-protein complexes and can coordinate chromatin remodeling through dual mechanisms: directly demethylating H3K27me3 and recruiting additional chromatin modifiers, such as histone acetyltransferases, to synergistically activate transcription [85]. The dysregulation of this fundamental derepression mechanism underlies diverse pathologies. UTX is frequently mutated in cancers like leukemia, where it functions as a classic tumor suppressor [86], yet it can be co-opted as an oncogenic coactivator for estrogen receptor (ER) in breast cancer (BCa) [87]. Its Y-chromosome homolog, UTY, shares structural similarity but possesses significantly reduced catalytic activity, highlighting the importance of specific residues for enzymatic function [69].

### JARID1 subfamily and JARID2

The JARID1 subfamily functions as erasers of the active transcription-associated marks H3K4me2/3, thereby playing a central role in transcriptional repression [88]. Their core catalytic mechanism directly opposes the function of SET1/MLL-family methyltransferases [89]. While JARID1A-D share this conserved demethylase activity, they exhibit distinct non-catalytic domains and protein interaction motifs that confer unique targeting specificities and functional roles within the cell [90, 91]. In stark contrast, JARID2 lacks key catalytic residues and is enzymatically inactive. Its primary molecular function is non-catalytic scaffolding within the PRC2, where it is crucial for the proper recruitment and enzymatic activity of the complex, facilitating the deposition of H3K27me3 [64, 65, 92, 93]. This demonstrates how the JMJD family includes members that have evolved to regulate chromatin primarily through structural roles rather than demethylase activity.

### JMJD4, JMJD5, JMJD6, and JMJD8

Apart from the aforementioned families, several JMJD members exhibit specialized or multifunctional roles that expand the regulatory scope of the JMJD family. JMJD4 functions exclusively as a lysyl hydroxylase, targeting translation termination factor eRF1, a mechanism distinct from histone demethylation [40]. JMJD5 is involved in metabolic regulation through interactions with enzymes like PKM2, influencing both cellular metabolism and nuclear gene transcription [94–96]. JMJD6 possesses remarkable dual lysyl-hydroxylase and arginine demethylase activities, allowing it to regulate diverse processes like transcription elongation and RNA splicing by modifying both histone (*e.g.,* H4R3me2) and non-histone substrates [48]. Furthermore, JMJD8 exhibits non-histone demethylase activity against AKT1 to modulate kinase signaling [50, 51].

### Regulation of JMJD demethylases by PTMs

Beyond their roles as epigenetic modifiers, the activity, stability, and localization of JMJD demethylases are precisely controlled by PTMs, forming an essential upstream regulatory layer. Phosphorylation rapidly modulates their activity and interactions. For example, PKA phosphorylates JMJD1A to promote its co-activator function [72, 73], while ERK-mediated phosphorylation stabilizes JMJD2B to support survival under metabolic stress [80]. Notably, phosphorylation also primes JMJD2B for recognition and degradation by the F-box protein 22 (Fbxo22)-containing SCF ubiquitin ligase complex [97]. Ubiquitination itself is a central regulator of JMJD protein homeostasis and function. It can drive degradative turnover, as exemplified by the cullin 4B (CUL4B)-DNA damage-binding protein 1 (DDB1)-constitutive photomorphogenic 1 (COP1) E3 ligase complex targeting UTX [98]. In contrast, non-degradative ubiquitination can directly modulate protein function and localization. A prime example is the HECT domain-containing E3 ubiquitin ligase 1 (HUWE1)-mediated ubiquitination of JMJD1A, which facilitates its recruitment to chromatin and enhances its role in the DNA damage response [99]. Other modifications like SUMOylation (*e.g.,* on JMJD2A) also provide additional layers of regulation, which critically switches its function to promote gene transcription [100]. This multifaceted regulatory network ensures that JMJD proteins are dynamically and precisely tuned in response to diverse physiological and pathological signals. To provide a consolidated overview of this multifaceted regulatory landscape, Table 1 summarizes the key enzymatic activities, primary functions, associated modifying enzymes, and essential residues for representative JMJD family members.

**Table 1:** Summary of the JMJD protein family: members, modifying enzymes, and functional outcomes

JMJD Protein	PTMs Type	Enzyme/Complex	Functional outcome
JMJD1A	Phosphorylation at S265 [72, 73] Ubiquitination at K918 [99] Phosphorylation [101] Phosphorylation [102]	PKA HUWE1 ACK1 JAK2	Activates thermogenic gene transcription (demethylase-independent) Enhances DNA repair gene expression; sensitizes cells to PARP inhibitors Confers tamoxifen resistance in breast cancer Enables STAT3 coactivation and promotes oncogenesis
JMJD2A	SUMOylation at K471 [100]	Viral K-bZIP	Activates KSHV viral gene transcription and replication
JMJD2B	Phosphorylation at T305, S352, S566 and T1065 [80] Ubiquitination [97]	ERK Fbxo22-SCF complex	Stabilizes JMJD2B to promote cell proliferation and chemoresistance Mediates degradation essential for tamoxifen response
JMJD2D	Catalytic residues H192/S200 [32, 79] Ubiquitination [103]	TRIM14	Mutation abolishes demethylase function Prevents autophagic degradation and enhances pro-inflammatory responses
JMJD3	Phosphorylation at T1044 [104]	PKA	Promotes nuclear translocation and hepatic lipid degradation
UTX	Ubiquitination [98]	CUL4B-DDB1-COP1 complex	Degradation of UTX promotes cancer progression

Abbreviations: *ACK1* activated Cdc42-associated kinase 1, *CUL4B* cullin 4B, *DDB1* DNA damage-binding protein 1, *COP1* Constitutive photomorphogenic 1, *ERK* extracellular signal-regulated kinase, *Fbxo22* F-box protein 22, *HUWE1* HECT domain‐containing E3 ubiquitin ligase 1, *KSHV* Kaposi’s sarcoma-associated herpesvirus, *PARP* poly(ADP-ribose) polymerase, *PKA* protein kinase A, *PTMs* post-translational modifications, *SCF* skp1-cul1-F-box protein complex, *STAT3* signal transducer and activator of transcription 3, *SUMOylation* small ubiquitin-like modifier conjugation, *TRIM14* tripartite motif-containing 14

In summary, the JMJD family exerts broad biological influence through a conserved catalytic core that has evolved for precise substrate targeting, establishes them as specific and indispensable nodes within complex regulatory networks. Their activities are further refined and dynamically integrated into cellular signaling by upstream inputs, primarily via PTMs. Consequently, dysregulation of these intricately regulated mechanisms, whether through altered expression, mutation, or aberrant control, disrupts epigenetic and signaling homeostasis. Thus, this homeostatic imbalance directly constitutes the basis for their pathogenic roles across a wide range of diseases.

## Pathogenesis of JMJDs in malignant tumors

As well-established epigenetic regulators, JMJD proteins are frequently dysregulated in human cancers. Epigenetic alterations are now recognized as a key driver of oncogenesis, governing the transcriptional programs and malignant phenotypes of transformed cells [105–108]. The JMJD family of histone demethylases plays a pivotal role in this process by dynamically erasing methylation marks on histones H3K4, H3K9, H3K27, and H3K36, thereby modulating the expression of oncogenes, tumor suppressors, and genes involved in core cancer hallmarks [17, 20]. This section provides a summary of the well-characterized, and often context-dependent, roles of distinct JMJD family members across major cancer types. We detail their mechanisms in tumor initiation, progression, therapy resistance, and metastasis, focusing on their regulation of key transcription factors and signaling pathways.

### JMJDs and prostate cancer (PCa)

The JMJD family of histone demethylases plays diverse but converging roles in PCa progression. Among them, JMJD1A has emerged as a central epigenetic driver of PCa progression by integrating transcriptional, post-translational, and DNA repair regulatory mechanisms. JMJD protein family contains a characteristic LXXLL motif that mediates interaction with nuclear receptors, particularly the androgen receptor (AR) [30, 109]. AR, a member of the steroid nuclear receptor superfamily, functions through a well-defined structural organization including an N-terminal transactivation domain, a DNA-binding domain, a hinge region, and a ligand-binding domain [110]. In PCa, JMJD1A knockdown significantly reduces AR expression and diminishes its recruitment to key enhancers such as c-Myc [71], underscoring JMJD1A as a pivotal co-regulator of AR signaling. Upon ligand binding, AR translocates into the nucleus and promotes the transcription of genes that drive proliferation and survival of PCa cells [71]. JMJD1A reinforces this process by erasing repressive H3K9me2 marks, thereby enhancing chromatin accessibility and facilitating AR-mediated transcriptional activation of canonical targets such as NKX3.1, PSA, and TMPRSS2 [30], all of which are critical players in prostate biology and carcinogenesis. Under hypoxic conditions, JMJD1A further cooperates with hypoxia-inducible factor-1 alpha (HIF-1α) and the acetyltransferase p300 to promote enhancer activation, highlighting its role as an integrator of androgen and hypoxia signaling [111]. In castration-resistant prostate cancer (CRPC), JMJD1A acquires additional functions by associating with the splicing factor heterogeneous nuclear ribonucleoprotein F (HNRNPF) to promote the inclusion of cryptic exons within the AR gene, leading to the generation of androgen receptor splice variant 7 (AR-V7), a constitutively active variant that drives resistance to androgen deprivation therapies [112, 113].

Furthermore, JMJD1A exerts profound influence over the oncogene *c-Myc*, which represents a critical downstream effector in PCa. At the transcriptional level, JMJD1A activates c-Myc transcription by demethylating H3K9me2 at the enhancer region and promoting AR recruitment, thereby enhancing c-Myc binding to the promoters of its downstream targets CDKN1A (p21) and CDKN2B (p15), and ultimately inhibiting PCa cell proliferation [71]. It is worth noting that the levels of JMJD1A in the cytoplasm and nucleus are comparable [114]. In addition to promoting transcription, JMJD1A stabilizes c-Myc by preventing HUWE1-mediated K48-linked ubiquitination and proteasomal degradation, a demethylase-independent mechanism that is particularly evident in AR-negative PCa cells such as PC3 and DU145 [71]. Functionally, restoration of c-Myc expression rescues proliferative defects in JMJD1A-depleted PCa models, both in vitro and in xenografts, establishing c-Myc as a key mediator in the oncogenic activity of JMJD1A. And JMJD1A orchestrates DNA damage responses by directly binding to promoters of DNA repair genes such as *XRCC6*, *PRKDC*, and *BARD1*, all of which are also c-Myc transcriptional targets [99, 115]. By removing repressive H3K9me2 marks, JMJD1A enhances c-Myc occupancy at these loci and promotes repair of DNA double-strand breaks. JMJD1A depletion delays the resolution of γ-H2AX foci following ionizing radiation, sensitizing PCa cells to both irradiation and poly(ADP-ribose) polymerase (PARP) inhibition. Mechanistically, JMJD1A undergoes HUWE1-mediated noncanonical ubiquitination at lysine 918 (K918) via K27/K29 linkages, which facilitates p300 recruitment and transcriptional activation of DNA repair programs [99]. However, mutation of the K918 site compromises the ability of JMJD1A to support DSB repair and significantly sensitizes PCa cells to PARP inhibitors [99]. Besides, JMJD1A also stabilizes Gli1, the transcription factor downstream of Hedgehog signaling, protecting it from Itch/Numb-mediated degradation, thereby extending its half-life and enhancing oncogenic signaling [116, 117]. Pharmacological inhibition of JmjC proteins destabilizes Gli1 and suppresses PCa cell proliferation [116], supporting the therapeutic relevance of targeting JMJD1A.

Beyond JMJD1A, the JMJD2 subfamily also participate in shaping PCa biology, particularly through their influence on AR signaling, chromatin remodeling, and therapy resistance. JMJD2A, which is frequently amplified in CRPC, has been found to promote castration resistance by activating AR enhancers through H3K4me2 demethylation and directly upregulating the expression of AR [118]. Besides, JMJD2A also suppresses the innate immune cGAS-STING pathway, facilitating cancer progression by impairing antitumor immunity [118]. Oncogenic collaboration between JMJD2A and ETS variant 1 (ETV1) activates pathways such as the Hippo-YAP1, thereby driving prostate tumorigenesis [119]. JMJD2A also confers docetaxel resistance by regulating the miR-34a-STMN1-β3-tubulin axis, linking epigenetic reprogramming with cytoskeletal remodeling and drug resistance [120]. However, the roles of JMJD2C and JMJD2D are less defined in PCa, which may be due to the possibility of sharing overlapping functionalities within this subfamily. Another set of demethylases relevant to PCa progression is the KDM6 family, including JMJD3 and UTX, which are commonly upregulated in CRPC [121]. Both JMJD3 and UTX enhance AR signaling by reducing the repressive H3K27me3 mark, thereby facilitating sustained transcriptional activation [122, 123]. Although JMJD3 and the methyltransferase EZH2 possess opposing enzymatic activities, they can cooperatively drive CRPC progression by regulating complementary oncogenic processes, including metabolic reprogramming and DNA repair [124–126]. Specifically, JMJD3 modulates the expression of genes critical for DNA repair (*e.g., MGMT*) and RNA splicing (*e.g., U2AF1* and *TRA2A*), further contributing to tumor aggressiveness [126]. Importantly, pharmacological inhibition of JMJD3 or UTX with small molecule GSK-J4 suppresses AR-mediated transcription and exhibits antitumor efficacy in CRPC models [121, 127], highlighting the translational potential of targeting this axis for therapeutic intervention.

JMJD5 and JMJD6 represent alternative layers of epigenetic regulation with unique connections to PCa metabolism and RNA splicing. JMJD5 functions as a metabolic integrator by coactivating PKM2, thereby coupling glycolytic reprogramming with AR-dependent transcription in advanced disease [94]. Of note, the co-activation of AR by JMJD5 does not rely on its demethylase activity toward H3K36me3 [94]. This may be attributed to its newly discovered proteolytic activity, which could modulate chromatin structure independently of its demethylase function, thereby facilitating AR recruitment to its target sites [45, 128]. JMJD6 has been previously reported to interact with numerous proteins involved in RNA processing [47, 129–131], including the splicing factor U2 small nuclear ribonucleoprotein auxiliary factor 65-kilodalton subunit (U2AF65), which is lysyl-5-hydroxylated by JMJD6 at residues within its arginine-serine-rich domain [47]. Besides, JMJD6 promotes the aberrant splicing of AR-V7 and drives castration-resistant progression in PCa through this hydroxylation of U2AF65 [132]. Notably, this catalytic activity can be blocked by the small molecule 2,4-PDCA, positioning JMJD6 as another promising druggable target within the JMJD network [132].

The JARID1 subfamily, which primarily demethylates H3K4me2/3, adds further complexity to the epigenetic landscape of PCa. JARID1B enhances AR activity by demethylating H3K4me3 at AR target genes, thereby reinforcing oncogenic androgen signaling [133]. Similarly, JARID1A is often overexpressed in PCa and contributes to tumor progression [134]. By contrast, JARID1D, a Y chromosome-encoded demethylase, is frequently downregulated in PCa [135, 136]. The loss of JARID1D correlates with heightened AR activity and therapeutic resistance, while its re-expression suppresses AR signaling and restores chemosensitivity [136], suggesting tumor-suppressive properties that are contextually opposed to other family members.

**Fig. 3:**
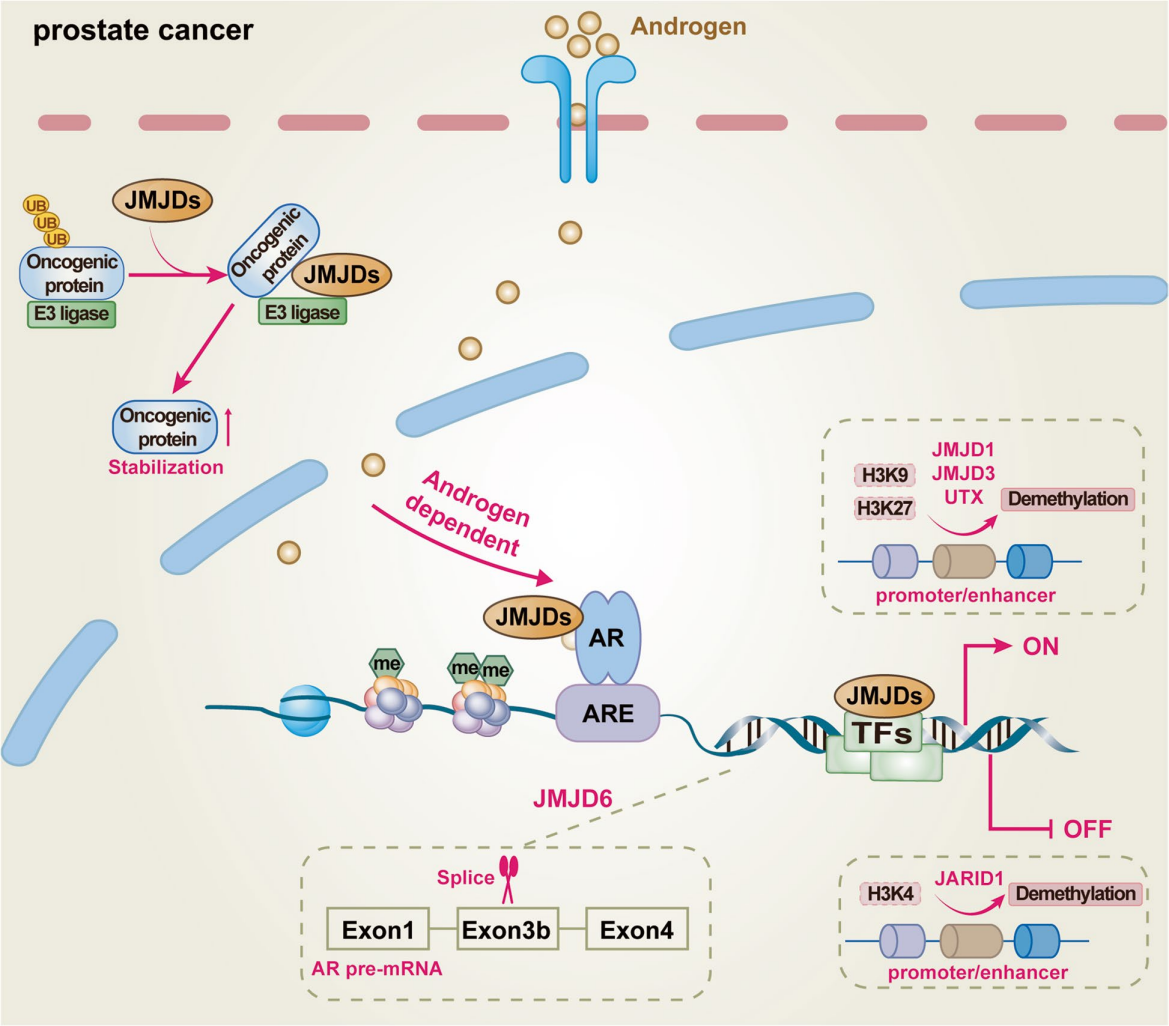
Schematic diagram illustrating the mechanistic basis of JMJD family proteins in PCa. The model highlights how members such as JMJD1A, UTX, and JMJD3 activate oncogenic transcription by erasing repressive histone marks (H3K9me2/3, H3K27me3), thereby sustaining AR signaling and related gene expression. In contrast, JARID1 family members repress tumor-suppressive pathways by demethylating the active H3K4me3 mark. This coordinated epigenetic reprogramming is further amplified through non-enzymatic mechanisms, including the regulation of oncoprotein stability (*e.g.,* preventing c-Myc ubiquitination and stabilizing Gli1) and the facilitation of AR pre-mRNA splicing (*e.g.,* promoting the generation of the AR-V7 variant through interactions with splicing factors). Abbreviations: me, methylation; ARE, androgen response element; AR, androgen receptor; TFs, transcription factors

Taken together, the JMJD family orchestrates a complex epigenetic network that drives PCa progression. JMJD1A serves as a master regulator coordinating AR signaling, c-Myc stabilization, DNA repair, and Hedgehog pathway activation, while other JMJD proteins extend these regulatory axes by influencing RNA splicing, metabolism, chromatin states, and therapy resistance. The multifaceted mechanisms by which key JMJD family members drive PCa progression are integrated into a simplified model in Fig. 3. Their overlapping yet non-redundant roles underscore the complexity of epigenetic regulation in this disease and reveal multiple therapeutic opportunities.

### JMJDs and BCa

Substantial clinical evidence indicates that the expression profiles of JMJD family members are significantly altered in BCa compared to those of normal breast tissue, and these alterations are closely associated with tumor subtypes, malignant progression, and patient prognosis [137]. By interfering with key biological processes such as ER signaling, cellular metabolism, DNA damage response, cell stemness, and metastasis, they constitute a complex epigenetic regulatory network that drives breast carcinogenesis, therapy resistance, and recurrence.

Clinical investigations have revealed that elevated expression of JMJD1A is associated with a more than threefold higher risk of mortality in patients with BCa [138]. JMJD1A exerts multifaceted regulatory effects on BCa pathogenesis through both epigenetic remodeling and interactions with non-histone proteins. During early malignant transformation, JMJD1A upregulation drives tumorigenesis by directly activating key oncogenes like *MYC* and *PAX3* through H3K9me2 demethylation [139]. Given that approximately two-thirds of BCa are ER-positive (ER^+^), the ER signaling pathway is a pivotal oncogenic driver in this malignancy [140]. Upon binding estrogen, ER recognizes estrogen response element (ERE) located within the promoters of target genes, initiating transcriptional programs that promote tumor cell proliferation and survival [140]. The primary therapeutic strategy for ER^+^ BCa involves endocrine therapy aimed at blocking ER-estrogen interactions; however, the emergence of therapeutic resistance poses a significant clinical challenge [141]. JMJD1A functions as a co-activator of ER by catalyzing the demethylation of H3K9me1/2, thereby enhancing ER recruitment to chromatin and facilitating the transcription of ER target genes critical for BCa progression [142]. JMJD1A knockdown not only reduces ER binding to the proximal promoters of genes like *pS2* and *GREB1* but also disrupts its occupancy at distal enhancers of genes, such as *CCND1*, *MYC*, and *XBP1* [142]. The failure of a catalytically inactive JMJD1A mutant to rescue ER target gene expression underscores the indispensability of its demethylase activity. Notably, in human epidermal growth factor receptor 2 (HER2)-overexpressing BCa, the activated Cdc42-associated kinase 1 (ACK1) phosphorylates and activates JMJD1A, enabling it to drive the expression of the ER-target oncogene homeobox A1 (*HOXA1*) to confer tamoxifen resistance [101]. Consequently, JMJD1A dependency persists in endocrine-resistant models, where its silencing effectively suppresses ER signaling and curbs cell proliferation, positioning JMJD1A as a promising therapeutic target for both treatment-naive and resistant ER^+^ disease [142].

Interestingly, JMJD1A displays context-dependent dual roles in BCa metastasis. In low- or moderate-metastatic BCa cells, JMJD1A promotes the transcription of pro-apoptotic genes (*BNIP3*, *BNIP3L*) via H3K9me2 demethylation, inducing anoikis and limiting metastasis [143]. In contrast, in highly invasive cells, JMJD1A adopts a pro-metastatic role by activating a repertoire of invasion-promoting genes including *MMP9*, *S100A4*, and *JUN* [144]. This functional duality highlights the remarkable plasticity of JMJD1A in determining tumor cell fate. In addition, JMJD1A contributes to chemoresistance by modulating the p53 signaling pathway. By demethylating the non-histone protein p53 at lysine 372 (K372), JMJD1A suppresses p53-mediated transcription of pro-apoptotic genes. Knockdown of JMJD1A increases levels of monomethylated p53 (K372me1), enhancing p53 binding to the promoters of PUMA and NOXA, and subsequently triggering apoptosis [144]. The abolition of this pro-apoptotic effect upon p53 co-silencing confirms the dependence of the mechanism on functional p53. Beyond transcription, JMJD1A also regulates the DNA damage response by influencing the alternative splicing of cell-cycle genes such as *SAT1* in a demethylase-independent manner [145]. Furthermore, JMJD1A contributes to the maintenance of cancer stem cells (CSCs), which are closely linked to tumor invasiveness, metastasis, and therapeutic resistance [146, 147]. While wild-type p53 typically constrains CSCs self-renewal, it is reversed in mutant p53 [148, 149]. Intriguingly, JMJD1A knockdown impairs tumorsphere formation and suppresses tumor growth in vivo [144]. In p53-mutant backgrounds, JMJD1A knockdown partially restores p53 transcriptional activity, induces expression of its target PUMA, and consequently sensitizes CSCs to chemotherapeutic agents. Beyond JMJD1A, its paralog JMJD1B can be co-opted by oncogenic structural variations. The JMJD1B-eRF1 fusion gene functions as a potent driver of metastatic progression in invasive ductal carcinoma [150]. It promotes invasion and metastasis by directly downregulating the expression of the tumor suppressor LIM domain only 2 (LMO2), which in turn leads to activation of the pro-invasive Wnt/β-catenin signaling pathway [150].

The JMJD2 subfamily predominantly acts as oncogenes in BCa, with their expression exhibiting distinct subtype specificity. JMJD2B overexpression is more dominant in ER^+^ luminal BCa, whereas JMJD2A, JMJD2C, and JMJD2D are significantly overexpressed and amplified in basal-like BCa [151]. The functionality of JMJD2B is particularly complex. It is not only a direct ERα target gene but also forms a positive feedback loop with ERα, potently co-activating ERα-mediated transcription. This is achieved by recruiting the mixed-lineage Leukemia 2 (MLL2) complex and the SWI/SNF chromatin remodeling complex, thereby coordinating the removal of H3K9me3 and the addition of H3K4me3 to drive luminal BCa proliferation [152, 153]. Furthermore, JMJD2B integrates hypoxic signaling, promoting cancer cell growth under both normoxic and hypoxic conditions by regulating cell cycle genes like *CCND1* [154]. In PTEN-deficient triple-negative breast cancer (TNBC), JMJD2B sustains cancer cell survival by interacting with eukaryotic translation initiation factor 2 alpha (eIF2α) in the cytoplasm to suppress the unfolded protein response (UPR), representing a therapeutic vulnerability that can be exploited in combination with phosphoinositide-3-kinase (PI3K) inhibitors [155]. Crucially, the stability of the JMJD2B protein itself is regulated post-translationally. The Fbxo22 E3 ubiquitin ligase complex targets JMJD2B for proteasomal degradation upon tamoxifen binding to ERα [97]. This degradation is essential for the release of transcriptional co-activators (*e.g.*, SRC-3) from ERα, which is a critical step in establishing the antagonistic effects of tamoxifen. Consequently, loss of Fbxo22 leads to JMJD2B stabilization, continued co-activator recruitment, and tamoxifen resistance, identifying both Fbxo22 and JMJD2B as key determinants of selective estrogen receptor modulators (SERMs) activity. Paradoxically, in certain contexts, JMJD2B also exhibits tumor-suppressive functions. Its downregulation promotes BCa cell stemness and metastasis by upregulating the metabolic gene phosphoglycerate dehydrogenase (*PHGDH*) via increased H3K36me3 enrichment on its promoter [156]. Separately, a circular RNA variant derived from the JMJD2B locus, circJMJD2B, functions as a molecular sponge for miR-675, leading to the upregulation of NEDD4L, subsequent degradation of PI3KCA, and inhibition of the PI3K-AKT pathway and angiogenesis, thereby exerting tumor-suppressive effects [157]. JMJD2A functions as a potent oncogenic driver in BCa by concurrently activating pro-tumorigenic pathways and repressing tumor suppressors. For example, JMJD2A can epigenetically activates the Notch1 signaling [158] and represses the Sp1-DIRAS3 tumor suppressor axis to promote metastasis [159]. Additionally, JMJD2A represses the tumor suppressor bone morphogenetic protein 9 (BMP9) to promote glutamine metabolism [81]. This repression operates through a dual mechanism: epigenetic silencing via H3K36 demethylation at the BMP9 promoter and reduction of BMP9 protein stability. Notably, the ensuing enhancement of glutamine metabolism leads to increased levels of 2-OG, which not only serves as an essential cofactor for demethylase activity of JMJD2A but also actively promotes the nuclear translocation of JMJD2A [81]. This creates a powerful feed-forward loop, further amplifying the oncogenic transcriptional program of JMJD2A. JMJD2C serves as a specific co-activator for HIF-1α, removing H3K9me3 from HIF target genes and potently activating genes involved in metabolic reprogramming (*e.g., LDHA*, *PDK1*) and metastasis (*e.g., LOXL2, L1CAM*), thereby driving tumor growth and lung metastasis [160]. Furthermore, JMJD2C can inhibit tumor growth through a non-canonical, demethylase-independent mechanism by promoting cathepsin L-mediated cleavage of histone H3, resulting in decreased expression of antioxidant enzymes and accumulation of reactive oxygen species [161].

The role of JMJD5 extends beyond traditional histone modification. It is induced by hypoxia and directly interacts with the metabolic enzyme PKM2, inhibiting its tetramerization and enzymatic activity while promoting its nuclear translocation [95]. In the nucleus, JMJD5, PKM2, and HIF-1α form a complex that co-activates the transcription of glycolysis-related genes (*e.g., LDHA*), reprogramming cancer cell metabolism to adapt to the hypoxic environment [95]. JMJD6 is a multifunctional protein whose overexpression is a robust marker of tumor aggressiveness and poor prognosis [162, 163]. It cooperates with the oncoprotein c-Myc via a self-reinforcing positive feedback loop. By demethylating H4R3me2a on the p19ARF promoter, JMJD6 suppresses the p53 pathway, overcoming Myc-induced apoptosis and driving tumorigenesis and metastasis [164]. Furthermore, JMJD6-mediated transcriptional programs lead to constitutively high levels of cyclin E1 and an inverse correlation with the transforming growth factor-beta (TGF-β) tumor suppressor pathway, particularly TGF-β2, consequently fostering a hyper-proliferative state [163]. Beyond its classical arginine demethylase activity, JMJD6 possesses an intrinsic tyrosine kinase activity, phosphorylating histone H2A variant X (H2A.X), which in turn promotes TNBC growth by inducing autophagy, providing a rationale for the combined inhibition of JMJD6 and autophagy [165]. JMJD10 is a JmjC domain-containing protein whose expression is increased in about 30% of BCa samples [166]. High JMJD10 expression is significantly associated with shorter overall survival, distant metastasis-free survival, and relapse-free survival, identifying it as an important prognostic factor for poorer survival [166].

The JARID1 subfamily demethylates H3K4me3, an activity generally associated with gene repression. JARID1B (also known as PLU-1) is a key driver of the luminal lineage. Its amplification and overexpression are crucial for maintaining luminal cell identity, as its knockdown induces basal gene expression and growth arrest [167]. In its role as a transcriptional repressor, JARID1B targets and silences multiple tumor suppressor genes, including the DNA repair factor *BRCA1* [91]. It can also form a complex with LSD1/NuRD to coordinately repress genes including the chemokine *CCL14*, thereby suppressing angiogenesis and metastasis in BCa [168]. Conversely, JARID1A (also known as RBP2) is a critical mediator of BCa progression and therapy resistance. It promotes metastasis as a pleiotropic regulator of many metastasis-associated genes [169]. Mechanistically, part of its pro-metastatic function is achieved by epigenetically activating the transcription of tenascin C (TNC), an extracellular matrix protein that creates a favorable microenvironment for invasion and metastasis [169]. Furthermore, JARID1A contributes to tamoxifen resistance by forming a complex with ER and histone deacetylase 1 (HDAC1) to epigenetically silence insulin-like growth factor binding protein 4/5 (IGFBP4/5), leading to insulin-like growth factor 1 receptor (IGF1R) activation. It also stabilizes ErbB proteins independently of its demethylase activity, enhancing IGF1R-ErbB crosstalk and subsequent PI3K-AKT activation [170]. JARID1A is also a significant therapeutic target. Small-molecule inhibitor cyclopenta[c]chromen derivative 1 [171] and a rhodium(III) complex [172] targeting JARID1A have been shown to inhibit its activity, leading to H3K4me3 accumulation and upregulation of cell cycle inhibitors like p27, consequently inducing cell cycle arrest and senescence, with significant anti-tumor effects in preclinical models.

The function of UTX is also context-dependent. In ER^+^ BCa, UTX forms a positive feedback loop with ER [87]. It coordinates H3K27 demethylation (catalyzed by itself) and histone acetylation (by recruiting CBP) to create an open chromatin state, co-activating oncogenes like *CXCR4* and driving hormonally responsive carcinogenesis [87]. Conversely, in suppressing metastasis, UTX recruits LSD1 and HDAC1 to form a repressive complex that removes H3K4me2 and histone acetylation from the promoters of epithelial-mesenchymal transition (EMT) transcription factors (*e.g., SNAIL*, *ZEB1/2*) and transcriptional repressor *CDH1*, silencing EMT genes and inhibiting cancer stem cell properties and metastasis [173]. This cooperative model is a recurrent theme, as multiple JMJD family members often partner with coregulators such as LSD1 to enact context-specific transcriptional programs.

**Fig. 4:**
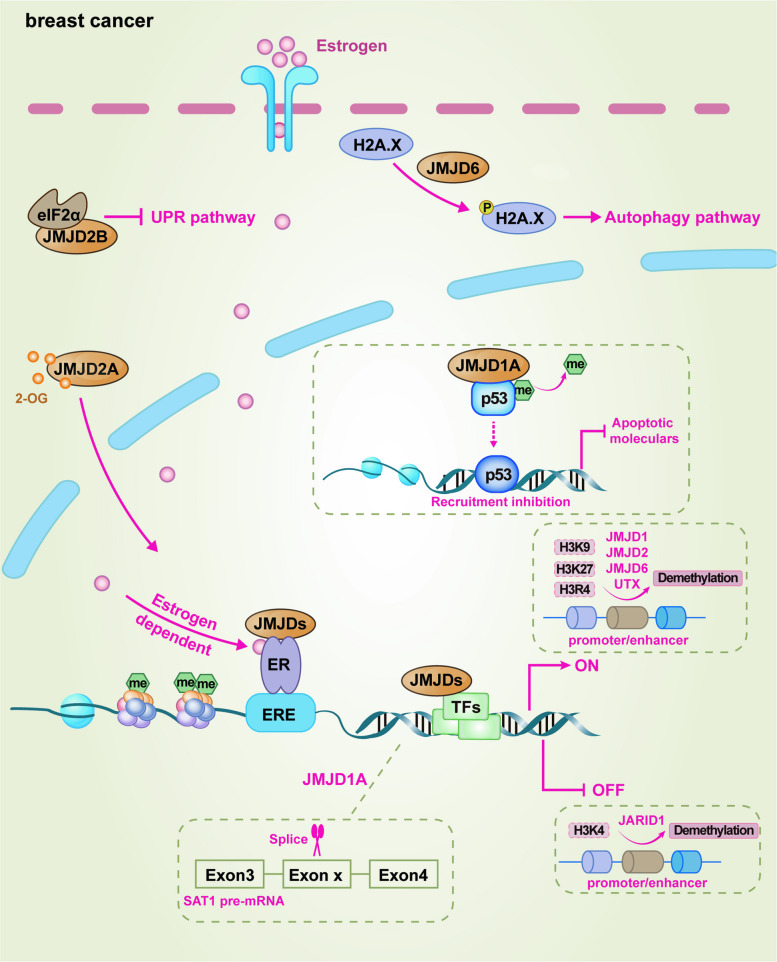
Schematic diagram illustrating the mechanistic basis of JMJD family proteins in BCa. The model highlights how members such as JMJD1, JMJD2, JMJD6, and UTX activate oncogenic transcription by removing methylation from H3K9, H3K27, and H4R3. In contrast, JARID1 family members repress tumor-suppressive pathways by demethylating the active H3K4me3 mark. This coordinated epigenetic reprogramming is further amplified through its nuclear translocation, modulated by intracellular 2-OG levels, and through direct intervention in the regulation of the UPR, autophagy pathways, and the alternative splicing of SAT1 pre-mRNA. Abbreviations: me, methylation; eIF2α, eukaryotic translation initiation factor 2 alpha; ER, estrogen receptor; ERE, estrogen response element; H2A.X, histone H2A variant X; H3R4, histone H3 arginine 4; SAT1, spermidine/spermine N1-acetyltransferase 1; UPR, unfolded protein response

The collective evidence establishes JMJD proteins as pivotal epigenetic orchestrators of BCa pathogenesis. The complexity and centrality of JMJD-regulated network are appreciated in the integrated schematic presented in Fig. 4.

### JMJDs and colorectal cancer (CRC)

The pathogenic landscape of CRC is profoundly shaped by the multifaceted functions of the JMJD protein family. Far beyond isolated enzymatic actions, these proteins form an intricate epigenetic web, coordinating a wide spectrum of oncogenic processes. Among them, JMJD1A stands out for its particularly well-characterized and multifaceted oncogenic roles. Its overexpression is frequently observed in clinical CRC samples and is positively correlated with the expression of key downstream target genes of the Wnt/β-catenin signaling pathway, including *c-Myc*, *cyclin D1*, and *MMP9*, implicating its pivotal role in CRC cell proliferation and metastasis [174]. Mechanistically, JMJD1A directly interacts with β-catenin to enhance its transcriptional activity while concurrently demethylating H3K9me2 at the promoters of target genes, thereby alleviating transcriptional repression. The indispensable role of its catalytic activity is highlighted by the failure of a catalytically inactive JMJD1A mutant (JMJD1A^H1120Y^) to promote CRC progression [174]. The functional significance of JMJD1A extends beyond the Wnt pathway, as it also serves as an essential factor for the activation of the JAK2-STAT3 signaling cascade, being phosphorylated by JAK2 and acting as a STAT3-dependent transcriptional coactivator to alter H3K9 methylation, positioning the inhibition of JMJD1A phosphorylation as a promising therapeutic strategy against this oncogenic pathway [102]. Beyond the Wnt and JAK-STAT signal pathway, JMJD1A promotes oncogenesis through other sophisticated mechanisms. Its collaboration with the chromatin remodeler alpha thalassemia/mental retardation syndrome X-linked (ATRX) synergistically promotes oncogenesis by cooperatively erasing H3K9me2 marks, as knocking down either factor significantly compromises CRC cell proliferation and viability [114, 175]. Furthermore, JMJD1A regulates the expression of Hippo signaling pathway-related genes through dual mechanisms, thereby participating in promoting tumorigenesis [176]. It directly enhances YAP1 transcription by demethylating H3K9me2 at its promoter, and simultaneously facilitates the expression of YAP1 downstream targets, such as *CTGF* and *CYR61*, by promoting enhancer activation via p300-mediated H3K27 acetylation [176]. This enhancer activation facilitates recruitment of the YAP1/TEA domain transcription factor 1 (TEAD1) transcriptional complex, amplifying downstream oncogenic signaling. Additionally, JMJD1A cooperates with the ETV1, being recruited to demethylate H3K9me2 at the promoters of oncogenic targets like *MMP1* and *FOXQ1*, thereby enhancing their transcription and facilitating tumor cell proliferation, invasion, and angiogenesis [177–179]. Under hypoxic conditions, JMJD1A further contributes to tumor progression by activating hypoxia-responsive genes such as adrenomedullin (*ADM*) and growth differentiation factor 15 (*GDF15*), supporting CRC cell survival and growth in the hypoxic tumor microenvironment (TME) [180]. The function of the JMJD1 family is further expanded by JMJD1C, which contributes to CRC metastasis by epigenetically regulating the expression of the transcription factor activating transcription factor 2 (ATF2) [181]. Beyond its role in oncogenesis, the JMJD1 family, particularly through JMJD1A and JMJD1B, is crucial for maintaining the tumorigenic potential of colorectal CSCs by epigenetically activating Wnt target gene transcription, positioning it as a key vulnerability in this therapy-resistant cell population [182].

Notably, JMJD2A exhibits remarkable context-dependent functionality. While it can promote cell survival and proliferation by acting as a transcriptional co-repressor for p53 target genes [183], it paradoxically exerts tumor-suppressive effects in immunotherapy contexts. This suppressive function is mechanistically linked to its ability to induce cellular senescence, a process initiated by the JMJD2A-driven suppression of the angiotensinogen (AGT)-prohibitin 1 (PHB1) axis, which consequently inhibits basal mitophagy [184]. This blockade leads to the accumulation of damaged mitochondria and cytoplasmic mitochondrial DNA, activating the cGAS-STING pathway and fostering a senescence-associated secretory phenotype (SASP) that enhances intratumoral CD8^+^ T cell infiltration and cytotoxicity [184]. JMJD2B serves as a multifunctional oncogenic hub. It functions as a co-activator for the Wnt/β-catenin pathway [185] and activates AKT signaling via a non-epigenetic interaction with TNF receptor-associated factor 6 (TRAF6), thereby reprogramming glucose metabolism [186]. Its role in promoting invasion is further exemplified by its ability to activate the transcription of the small GTPase TC10-like (TCL) by interacting with and being recruited by the transcription factor ETS-related gene 1 (ERG1) to the TCL promoter, facilitating pre-initiation complex assembly [187]. Under metabolic stress like glucose deprivation, JMJD2B is stabilized via ERK-mediated phosphorylation and subsequently upregulates LC3B to activate autophagy, sustaining amino acid pools and promoting cancer cell survival [80, 188]. The oncogenic role of JMJD2B is reinforced by its involvement in immune evasion, where it is part of the JMJD2B-HOXC4-PD-L1 axis targeted by tumor-suppressive microRNAs delivered via mesenchymal stem cell-derived extracellular vesicles [189]. Furthermore, JMJD2B enhances cell survival by transcriptionally activating the pro-survival gene *HAX1* to suppress mitochondrial apoptosis, thereby reinforcing its anti-cell death capabilities [190]. Interestingly, JMJD2B acts as a critical epigenetic bridge linking the oncogenic gut microbiota, specifically Enterotoxigenic *Bacteroides fragilis* (ETBF), to cancer stemness by upregulating core pluripotency factors like NANOG [191]. Conversely, JMJD2B depletion launches a coordinated multi-pronged attack on cancer cells, inducing apoptosis via concurrent mitochondrial and death receptor pathway activation [192]. This is coupled with a robust DNA damage response (DDR) mediated through ATM/ATR and enhanced by suppression of the STAT3 signaling pathway, leading to cell cycle arrest and senescence [193]. Crucially, the JMJD2B-STAT3-DDR axis profoundly sensitizes tumors to radiotherapy, underscoring its therapeutic relevance [194]. The in vivo efficacy of this approach is confirmed by significant tumor growth inhibition following JMJD2B knockdown [193]. The expression of JMJD2B is driven by multiple upstream signals, including the oncogenic gut microbiota ETBF via the TLR4-nuclear factor of activated T cells 5 (NFAT5) axis, and the transcription factor cAMP response element-binding protein (CREB), which directly binds to JMJD2B promoter to enhance its transcription [191, 194]. JMJD2D is another potent oncogene, driving tumorigenesis by directly activating Wnt/β-catenin signaling [79], employing a multi-tiered mechanism to activate the HIF-1 pathway and enhance glycolysis [195], and upregulating PD-L1 to facilitate immune evasion [196]. Its role extends to inflammation-driven tumorigenesis, where it is induced by TNFα and activates the Hedgehog signaling pathway [197].

The roles of JMJD3 and UTX, both targeting the repressive H3K27me3 mark, are complex and often contrasting in CRC. JMJD3 predominantly exhibits tumor-suppressive properties. Its low expression predicts unfavorable outcomes and it exerts its function partly by activating the cell cycle inhibitor p15INK4B [198]. Furthermore, JMJD3 is a key mediator of the anti-cancer effects of vitamin D, which signals through the vitamin D receptor (VDR) to directly activate JMJD3 gene transcription, facilitating the suppression of Wnt/β-catenin signaling and EMT [199, 200]. UTX, however, presents a paradigm of context dependency. It can promote proliferation by downregulating p21 and upregulating kinesin family member 14 (KIF14) [201], yet also function as a metastasis suppressor by orchestrating a chromatin state switch at the E-cadherin promoter, simultaneously demethylating H3K27me3 and recruiting CREB-binding protein (CBP) for H3K27 acetylation to activate transcription [202]. The stability of UTX itself is a regulated process, controlled by the CUL4B-DDB1-COP1 E3 ubiquitin ligase complex, and its degradation promotes CRC progression [98]. Intriguingly, loss of UTX initiates a unique tyrosine metabolic reprogramming cascade that fosters an immunosuppressive TME by enhancing myeloid-derived suppressor cells (MDSC) accumulation [203]. Importantly, the regulatory landscape of H3K27me3 is further complicated by JARID2, a JMJD family member that lacks catalytic demethylase activity. JARID2 functions as a crucial scaffold within the PRC2, enhancing its ability to deposit the repressive H3K27me3 mark. During TGF-β-induced EMT, JARID2 is upregulated and facilitates the recruitment of PRC2 to the promoters of key epithelial genes, such as *CDH1* and the miR-200 family, leading to their silencing and thereby driving CRC cell invasion and metastasis [93]. Other JMJD family members, though less extensively studied, are significantly implicated in CRC. JMJD4 is overexpressed in CRC and linked to poor prognosis, promoting tumor progression by inhibiting the PDCD5-p53 pathway [204, 205]. JMJD5 is identified as a potential oncogene, whose knockdown suppresses proliferation, migration, and invasion [206]. JMJD6 promotes carcinogenesis through a non-histone demethylase activity, hydroxylating p53 at lysine 382 to repress its transcriptional activity and acetylation [207]. JMJD8, an oncogene, is directly targeted and suppressed by the tumor-suppressive miR-873-5p, influencing CRC through the NF-κB pathway [208]. JMJD10, a c-Myc target gene, is highly expressed in CRC, and its nuclear localization serves as a critical indicator of poor prognosis in patients receiving adjuvant chemotherapy, essential for sustaining cancer cell proliferation [209, 210].

The JARID1 family, H3K4me3 demethylases, displays astonishing functional diversity in CRC. JARID1A can act as a tumor suppressor within a specific oncogenic pathway. Its expression is suppressed by the oncogenic lncRNA NEAT1, leading to the derepression of oncogene *Cul4A* and subsequent activation of the Wnt pathway [211]. In stark contrast, JARID1B is a well-established oncogene. It drives proliferation by transcriptionally silencing multiple tumor suppressors, including CDX2 and CCL14, thereby activating Wnt/β-catenin signaling [212, 213], and is crucial for maintaining proliferative capacity and resisting senescence [214]. JARID1C exemplifies functional pleiotropy. It promotes proliferation and metastasis by epigenetically repressing F-box/WD repeat-containing protein 7 (FBXW7) (leading to c-Jun accumulation) and METTL14 (leading to SOX4 mRNA stabilization) [215, 216]. Paradoxically, in a chemotherapy context, it suppresses multidrug resistance by repressing the drug efflux pump ATP-binding cassette subfamily C member 1 (ABCC1), thereby enhancing sensitivity to oxaliplatin and irinotecan [217]. It also regulates proliferation via the lncRNA HOXC-AS3 and its effect on DLG4 mRNA stability [218]. Perhaps the most striking is the role of JARID1D, which provides a genetic explanation for sex disparities in CRC. This Y chromosome-encoded gene is specifically upregulated by the KRAS-STAT4 axis in male-specific KRAS-mutant CRC, where it drives invasion and immune evasion through the direct repression of key effector genes [219]. It compromises epithelial integrity by targeting AMOT and impairs antigen presentation by silencing TAP1/TAP2, thereby facilitating metastasis and CD8^+^ T cell evasion [219]. However, the function of JARID1D is highly context-dependent, as another study in a broader male cohort identified a tumor-suppressive role mediated through its suppression of the E2F transcription factor 1 (E2F1)-FK506-binding protein 4 (FKBP4) axis [220], underscoring the pivotal influence of specific molecular subtypes.

**Fig. 5:**
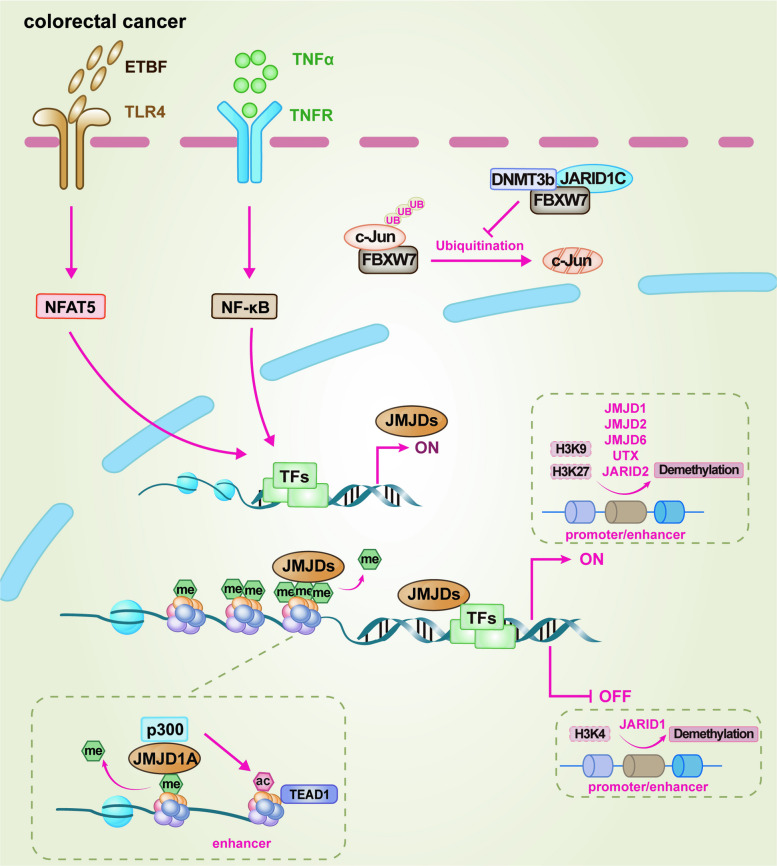
Schematic diagram illustrating the mechanistic basis of JMJD family proteins in CRC. The model highlights how specific members, including JMJD1, JMJD2, JMJD6, UTX, and JARID2, activate oncogenic transcription by removing methylation from H3K9 and H3K27. In contrast, JARID1 family members repress tumor-suppressive pathways by demethylating H3K4me3. This core epigenetic reprogramming is amplified by recruiting co-activators like p300 for enhancer acetylation, fine-tuning the transcriptional output of key pathways. Besides, JMJD protein expression and function are dynamically regulated by upstream oncogenic signals from the gut microbiota (*e.g.,* ETBF via the TLR4-NFAT5 axis) and the inflammatory tumor microenvironment (*e.g.,* TNFα via NF-κB), positioning the JMJD family as central integrators of extracellular cues that fuel the CRC pathogenic landscape. Abbreviations: ac, acetylation; me, methylation; DNMT3b, DNA methyltransferase 3 beta; ETBF, Enterotoxigenic *Bacteroides fragilis*; FBXW7, F-box/WD repeat-containing protein 7; NFAT5, nuclear factor of activated T cells 5; NF-κB, nuclear factor kappa-B; TEAD1, TEA domain transcription factor 1; TFs, transcription factors; TLR4, Toll-like receptor 4; TNFR, tumor necrosis factor receptor

Taken together, members of the JMJD family, through their diverse enzymatic activities and protein interactions, masterfully regulate diverse biological functions of CRC, including sustained proliferation, metabolic reprogramming, invasion and metastasis, immune evasion, and therapy resistance. The core molecular mechanisms through which representative JMJD family members drive CRC progression are delineated in Fig. 5. Their prominent roles underscore their potential as valuable prognostic biomarkers and promising therapeutic targets.

### JMJDs and hepatocellular carcinoma (HCC)

The JMJD family plays a multifaceted and critical role in the pathogenesis of HCC, governing essential processes from tumor initiation, progression, and therapeutic resistance. These enzymes, by dynamically regulating histone methylation landscapes, influence critical cancer hallmarks including cell cycle progression, metabolic reprogramming, stemness maintenance, and metastasis. The JMJD1 family, particularly JMJD1A and JMJD1B, has been implicated in tumor promotion. JMJD1A is upregulated in HCC and promotes proliferation under hypoxia by demethylating H3K9me2 at the ADM promoter and via the PI3K-AP-1 pathway [221, 222]. Meanwhile, JMJD1B also shows increased expression in HCC, and its ablation by CRISPR/Cas9 significantly retards the cell cycle and proliferation of HCC [223]. Approximately 30% of JMJD1B knockout cells exhibit mitotic spindle multipolarity, indicating a chromosome instability phenotype. Mechanistically, JMJD1B regulates a transcriptional network of cell-cycle genes, especially the cell proliferation factor *CDC123*. Additionally, JMJD1B knockout reduces Cyclin D1 protein levels through proteosomal degradation, suggesting a post-translational regulatory mechanism [223].

Members of the JMJD2 family are established oncogenes and promising therapeutic targets. *JMJD2A* has been identified as a major downstream target gene of the transcription factor regulatory factor X 5 (RFX5), which binds directly to the JMJD2A promoter region to promote its transcription [224]. This RFX5-JMJD2A pathway promotes cell cycle progression from G0/G1 to S phase and protects against cell apoptosis through the regulation of p53 and its downstream genes in HCC [224]. JMJD2C inhibition enhances radiosensitivity by increasing H3K36me3 on the CXCL2 promoter, upregulating its expression and impairing DNA repair [225]. The observed effects of JMJD2C depletion can be partially rescued by CXCL2 silencing, confirming CXCL2 as a key downstream effector. JMJD2D demonstrates particularly complex mechanisms, promoting liver cancer stem-like cell self-renewal by enhancing epithelial cell adhesion molecule (EpCAM) and SRY-box transcription factor 9 (SOX9) expression. It achieves this by reducing H3K9me3 levels on their promoters through interactions with β-catenin/TCF4 and Notch1 intracellular domain, respectively [226]. Additionally, JMJD2D exhibits a demethylase-independent function as a novel antagonist of p53, directly interacting with p53 and inhibiting its recruitment to the *p21* and *PUMA* promoters to promote HCC initiation and progression [227]. Notably, the therapeutic potential of targeting this family is underscored by findings that the JMJD2-specific inhibitor ML324 induces apoptosis through the UPR and Bim upregulation in HCC cells [228].

JMJD3 and UTX are implicated in invasion and microenvironment crosstalk. JMJD3 shows aberrant expression that upregulates Slug to promote migration, invasion, and stem cell-like behaviors by demethylating H3K27me3 at the Slug promoter [229]. It is also recruited by the lncRNA DNM3OS in a tumor-associated mesenchymal stem cell pathway to activate T-cell lymphoma invasion and metastasis 1 (TIAM1) expression, fueling metastasis [230]. However, the role of UTX is context-dependent. It primarily acts as an oncogene by upregulating fibroblast growth factor receptor 4 (FGFR4) to activate the PI3K-AKT-mTOR pathway and metabolic reprogramming, with its expression positively correlating with lenvatinib efficacy [231]. Paradoxically, one study identifies it as a tumor suppressor that negatively regulates the TGF-β-SMAD pathway [232], highlighting the complex biology of this family. The JMJD6 family demonstrates functional versatility. JMJD6 drives carcinogenesis by directly transactivating cyclin-dependent kinase 4 (CDK4) [233] and forms a complex with bromodomain-containing protein 4 (BRD4) to enhance *HOTAIR* transcription, fostering radioresistance in liver cancer stem cells [234], which was augmented by the c-Jun-JMJD6-IL-4-ERK pathway [235]. Recently, JMJD6 was found to complex with BRD4 to suppress peroxisome proliferator-activated receptor gamma (PPARγ) by reducing promoter H4R3me2s, activating GPX4 to confer ferroptosis resistance, which can be overcome with JMJD6 inhibitors [236].

The JARID1 family represents crucial regulators of cell cycle and drug response in HCC. JARID1A drives chemoresistance via the Rho-associated coiled-coil containing protein kinase 1 (ROCK1)-PTEN-AKT axis [237] and promotes angiogenesis by suppressing miR-433 to activate the FXYD domain-containing ion transport regulator 3 (FXYD3)-PI3K-AKT pathway [238]. The pharmacological inactivation of JARID1A with CPI-455 enhances the cytotoxicity of cisplatin, resulting in apoptosis and mitochondrial dysfunction [237]. JARID1B silences tumor suppressors p15 and p27 by removing H3K4me3 from their promoters [239] and sustains self-renewal via the microRNA-448-mediated YTH N6-methyladenosine RNA binding protein 3 (YTHDF3)-integrin subunit alpha 6 (ITGA6) axis [240]. Notably, the loss of axis inhibition protein 1 (AXIN1) regulates response to lenvatinib through the Wnt-JARID1B-p15 pathway, where JARID1B inhibition can upregulate p15 expression and increase sensitivity to Lenvatinib [241]. In contrast, JARID1D functions as a tumor suppressor by repressing the E2F1-TNNC1 axis [242]. Other JMJD family members also contribute significantly to HCC pathogenesis. JARID2, a PRC2 recruiter, silences PTEN by elevating H3K27me3 on its promoter, activating AKT and EMT to promote metastasis [243]. JMJD10, regulated by the IKAROS family zinc finger 1 (IKZF1)-MYC axis, fosters progression by demethylating H3K9me3 to repress p21 [244]. Furthermore, ZNF143-mediated H3K9 trimethylation upregulates cell division cycle 6 (CDC6) by activating JMJD10 in HCC, proposing a model for a ZNF143-JMJD10-CDC6 oncoprotein axis [245]. JMJD4 represents a unique family member that demethylates retinoic acid-inducible gene I (RIG-I) to prevent hepatic steatosis and carcinogenesis [246]. JMJD4-mediated RIG-I demethylation suppresses IL-6-STAT3 signaling, while constitutive methylated RIG-I associates with AMPKα to inhibit 3-hydroxy-3-methylglutaryl-CoA reductase (HMGCR) phosphorylation, thus promoting HMGCR enzymatic activity and cholesterol synthesis. This dual mechanism allows JMJD4-demethylated RIG-I to prevent both necroinflammation and non-alcoholic steatohepatitis (NASH)-induced hepatocarcinogenesis.

Briefly, the JMJD family constitutes a sophisticated epigenetic regulatory network that profoundly influences HCC biology. The context-dependent functions of certain family members underscore the complexity of epigenetic regulation in cancer and emphasize the necessity for precise patient stratification in future therapeutic approaches targeting these enzymes. As research continues to unravel the intricate mechanisms through which JMJD proteins control HCC progression, they present promising targets for novel epigenetic therapies that may overcome current limitations in HCC treatment.

### JMJDs and gastric cancer (GC)

In GC, members of the JMJD histone demethylase family play diverse and critical roles through distinct epigenetic mechanisms, significantly influencing tumorigenesis and progression. JMJD1A, in particular, exhibits context-dependent functions. On one hand, its upregulation is closely associated with enhanced tumor invasiveness, lymph node metastasis, advanced TNM stage, and poorer overall survival [247]. This oncogenic effect is mediated by reducing H3K9me2 levels at the metastasis-associated lung adenocarcinoma transcript 1 (MALAT1) promoter, leading to the transcriptional activation of this long non-coding RNA, which in turn promotes proliferation via the MAPK signaling pathway. On the other hand, JMJD1A cooperates with the transcription factor E26 transformation-specific 1 (ETS-1) to reduce H3K9me1/2 methylation at the runt-related transcription factor 3 (RUNX3) promoter, thereby upregulating RUNX3 expression, inhibiting proliferation, and reducing xenograft tumor growth by 60% [248]. Tissue microarray analysis further reveals a significant positive correlation between JMJD1A and RUNX3 expression [248], suggesting its role is highly context-dependent, potentially shaped by the TME.

JMJD2 subfamily generally demonstrate pro-tumorigenic properties in GC. JMJD2B physically interacts with β-catenin to enhance its nuclear localization and transcriptional activity, which enables their subsequent binding to the vimentin promoter and reduction of local H3K9me3 levels, thereby inducing EMT [249]. In the context of *H. pylori* infection, β-catenin directly transactivates JMJD2B, which then cooperates with NF-κB to promote gastric carcinogenesis by demethylating H3K9me3 at the cyclooxygenase-2 (COX-2) promoter [250]. Furthermore, JMJD2B acts as a coactivator for c-Jun, regulating the expression of genes including *IL-8*, *MMP1*, and *ITGAV* to foster cell migration and *H. pylori*-associated oncogenesis [251]. JMJD2A exerts its effect by enhancing chemosensitivity via stabilization of the pro-apoptotic protein coiled-coil domain containing 8 (CCDC8), and its overexpression is linked to better tumor regression in patients receiving docetaxel, cisplatin, and S-1 (DCS) therapy [252]. Conversely, another report indicates JMJD2A promotes cell proliferation by repressing the pro-apoptotic miR-34a and is associated with poor prognosis [253], highlighting potential context-specific functions. JMJD2C emerges as a key vulnerability in p53-mutant GC, where it helps cancer cells evade senescence by regulating the Sp1-CDK2 axis [254]. The JMJD2C inhibitor QC6352, combined with the senolytic agent SSK1, demonstrates significant efficacy in a “one-two punch” therapeutic strategy [254]. In GC stem cells, JMJD2C forms a feed-forward loop with aldehyde dehydrogenase 1 family member A3 (ALDH1A3) to maintain stemness, tumorigenicity, and chemoresistance [255]. Furthermore, JMJD2D promotes progression in gastrointestinal stromal tumor (GIST) by directly binding to the HIF-1β promoter, reducing H3K9me3/H3K36me3 levels, and activating the HIF-1β-VEGFA signaling axis to stimulate angiogenesis [256].

JMJD3 and UTX exhibits functional divergence in GC. JMJD3, induced by *H. pylori*, promotes tumorigenesis and metastasis through demethylating H3K27me3 at the CXCR4 promoter and resulting upregulation of CXCR4 [257]. JMJD3 functions as an independent prognostic factor, exerting oncogenic effects through both demethylase-dependent and independent mechanisms, and its inhibitor GSK-J4 can counteract these effects [258]. In contrast, UTX acts as a potent tumor suppressor. Its downregulation activates the Wnt/β-catenin signaling pathway, promoting EMT, and correlates with poor differentiation, advanced TNM stage, and unfavorable prognosis [259]. In GIST, UTX inhibits metastasis and reprograms the TME towards a less immunosuppressive state by transcriptionally activating secreted protein acidic and rich in cysteine like protein 1 (SPARCL1), which subsequently inhibits p65 nuclear translocation [260].

The JARID1 subfamily members are also pivotal regulators in GC networks. JARID1A is upregulated by *H. pylori* CagA via the PI3K-AKT-Sp1 pathway, driving malignant transformation of gastric epithelial cells through Cyclin D1 activation [261]. Its overexpression also enables escape from cellular senescence by repressing CDKIs such as p21, p27, and p16 [262]. In metastasis, JARID1A participates in a positive feedback circuit: TGF-β1 induces JARID1A via p-SMAD3, and JARID1A, in turn, is recruited by p-SMAD3 to suppress E-cadherin transcription, facilitating EMT and distant metastasis [263]. Additionally, JARID1A promotes tumor angiogenesis by repressing vascular endothelial growth factor (VEGF) expression through H3K4me3 demethylation at its promoter [264]. JARID1B expression is regulated by multiple mechanisms, including copy number amplification, DNA hypomethylation, transcriptional activation by Yin Yang 1 (YY1), and post-transcriptional repression by miR-29a-3p [265]. It confers chemoresistance by demethylating H3K4 to facilitate X-ray repair cross complementing protein 1 (XRCC1) recruitment to DNA damage sites for enhanced repair [266]. This process is stabilized by the molecular chaperone heat shock protein 90 (HSP90), and the HSP90 inhibitor 17-AAG can sensitize cells by inducing JARID1B degradation. In *H. pylori* infection, the bacteria downregulate miR-29c, alleviating its suppression of JARID1B and consequently promoting cell proliferation via Cyclin D1 upregulation [267]. Besides, JARID1A also contributes to tumor development within the ELK4-JARID1A-PJA2-KSR1 axis, which promotes M2 polarization of macrophages [268]. Moreover, JMJD10 shows progressively increased expression during gastric adenocarcinogenesis. It promotes GC by regulating the H3K9me3 status and activating the AP-1 signaling pathway to control cell cycle progression [54]. Its overexpression is a significant marker for diagnosis and poor prognosis [269].

Collectively, these findings elucidate a complex network wherein JMJD family members modulate the histone methylation landscape, so fundamentally influencing oncogenic signaling and cell fate in GC. The contrasting yet convergent impacts of key JMJD members on fundamental biological processes in HCC and GC highlight their shared and unique roles in cell cycle regulation, metastasis, therapy resistance, and tumor microenvironment remodeling.

### The role of JMJDs in other cancers

In addition to its well-characterized roles in the aforementioned cancer types, the JMJD family also plays pivotal and complex roles in the tumorigenesis and progression of other malignancies, most notably in leukemias and gliomas. The JARID1 subfamily is predominantly oncogenic in acute myeloid leukemia (AML). JARID1A is a promising therapeutic target, whose knockdown exerts potent anti-leukemic effects in AML cells [270]. This is further underscored by the aggressive nature of leukemias driven by the nucleoporin 98 (NUP98)-JARID1A fusion oncoprotein, which promotes leukemogenesis through mechanisms including aberrant *HOX* gene expression and induction of genomic instability [271–273]. While JARID1A acts as an oncogene in AML, it functions as a tumor suppressor in chronic myeloid leukemia (CML), where its downregulation promotes the transition from chronic phase to blast crisis through an RBP2-PTEN-BCR-ABL cascade [274]. This highlights that the oncogenic versus tumor-suppressive function of JARID1 proteins is not fixed but is determined by the specific cellular and genetic context. Its paralog, JARID1B, supports AML cell survival and proliferation through pathways like the miR-140-3p-BCL2 axis and, in a complex interplay with the PRC2 complex, can also sustain tumorigenicity in certain AML subtypes [275, 276]. Furthermore, JARID1B contributes to chemoresistance by stabilizing the DNA damage repair machinery in a WW domain-containing E3 ubiquitin protein ligase 1 (WWP1)-dependent manner [277]. In contrast, JARID1C functions as a tumor suppressor in AML by repressing bivalently marked immature genes to promote differentiation [278], and interestingly, it can also enhance sensitivity to the drug lenalidomide by stabilizing cereblon in an enzyme-independent manner [279].

Beyond the JARID1 subfamily, the JMJD1 and JMJD2 members are also vital for leukemia maintenance. JMJD1B displays a dual role, acting as an oncogene by activating LMO2 in acute lymphoblastic leukemia (ALL) [280], but showing tumor-suppressive activity in specific AML contexts, notably in subtypes with MLL-AF6/9 or PML-RARα rearrangements, as well as in acute promyelocytic leukemia (APL), where it facilitates differentiation and oncoprotein degradation [281, 282]. Besides, JMJD1B also regulates autophagy through GABA type A receptor‑associated protein (GABARAP) [283]. JMJD1C is a universal coactivator for key oncogenic transcription factors like RUNX1-RUNX1T1 and homeobox A9 (HOXA9), essential for leukemia stem cells (LSCs) self-renewal and survival in diverse AML subtypes [74, 75, 284, 285]. Its function can involve non-catalytic mechanisms like biomolecular condensate formation and the regulation of critical metabolic pathways such as lipid synthesis [75, 286]. The JMJD2 demethylases are frequently overexpressed and contribute to leukemogenesis through distinct programs. JMJD2A is essential for AML self-renewal via a polymerase-associated factor 1 (PAF1)-mediated network [287], while JMJD2B and JMJD2C are regulated by hypoxia and NFE2, respectively, and are required for cell survival in specific AML and myeloproliferative neoplasm contexts [288, 289]. JMJD2C also collaborates with protein arginine methyltransferase 1 (PRMT1) in an aberrant epigenetic circuit and promotes LSCs function through an ALKBH5-AXL signaling axis [290, 291]. Moreover, JMJD2D promotes AML development by activating myeloid cell leukemia 1 (MCL-1) [292].

The functional complexity of JMJD proteins is perhaps best exemplified by the KDM6 family. UTX is a well-established tumor suppressor in AML and chronic myelomonocytic leukemia (CMML), where its loss leads to aberrant epigenetic programs, chemoresistance, and sensitization to PARP and BCL2 inhibition [86, 293–295]. However, in specific T-cell acute lymphoblastic leukemia (T-ALL) subtypes, it is a pro-oncogenic cofactor for TAL1, which has prompted the consideration of its inhibition as a therapeutic strategy [296]. This functional dichotomy is mirrored by JMJD3. While it can exert oncorepressor activity in certain AML subtypes, particularly FAB-M2 and M3, by facilitating a C/EBPβ-centered differentiation program [297], it is a critical dependency in NOTCH1-driven T-ALL, where it protects cells from oncogene-induced stress [298]. This oncogenic role extends to cooperation with other mutations, such as TET2 deficiency and ASXL1 truncations, in the pathogenesis of CMML and other myeloid malignancies, making it a compelling therapeutic target [299, 300].

The functional complexity of JMJD proteins extends profoundly to glioblastoma (GBM), the most aggressive primary brain tumor, where various members orchestrate core aspects of malignancy. The JARID1 subfamily is a critical mediator of therapy resistance. JARID1A and JARID1B are both key drivers of temozolomide (TMZ) resistance. The expression of JARID1A is induced by TMZ in a reversible, epigenetic manner, and its inactivation can restore drug sensitivity [301]. JARID1B serves as a predictive biomarker, where its pre-existing high expression in specific subclones, particularly stem-like cells, dictates their expansion upon TMZ exposure by modulating the PTEN-AKT survival axis [302].

Beyond drug resistance, the JMJD2 subfamily is the master regulator of GBM pathogenesis. JMJD2A promotes glioma cell growth by activating the AKT-mTOR signaling pathway [303] and also contributes to TMZ resistance through the HUWE1-Rho-associated coiled-coil containing protein kinase 1 (ROCK2) axis [304]. JMJD2B acts as a potent oncogene by epigenetically stabilizing the MYC protein and activating cell cycle genes, thereby accelerating GBM progression [305]. Intriguingly, in a context-dependent manner, its inactivation can cooperate with ATRX mutations to drive the alternative lengthening of telomeres (ALT) pathway, showcasing a dualistic function [306]. Similarly, JMJD2C is a central node in oncogenic signaling, being stabilized by the Wnt pathway to co-activate β-catenin target genes [307] and executing a “dual-hit” mechanism by simultaneously activating c-Myc and suppressing the pro-apoptotic function of p53 [308].

The KDM6 family exemplifies striking functional dichotomies in GBM, heavily influenced by cellular context. JMJD3 can function as a tumor suppressor in glioblastoma stem cells (GSCs), where it is repressed by STAT3 and promotes differentiation and p53-mediated senescence [309, 310]. Conversely, in more differentiated glioma cells, JMJD3 can adopt an oncogenic role by promoting EMT and migration via the CXCL12-CXCR4 and SNAI1 axes [311, 312]. This complexity is further highlighted in the TME, where its inhibition in myeloid cells reverses immune suppression and sensitizes tumors to PD-1 blockade [313]. UTX also displays multifaceted roles, which acts as a cooperating oncogene with homeobox A3 (HOXA3) to drive aerobic glycolysis [314], and its inhibition suppresses GSCs growth by modulating periostin expression [315]. However, studies in neural development suggest its function is highly context-dependent, where it acts to suppress a gliogenic program and promote neuronal differentiation [316]. This developmental role as an inhibitor of glial cell fate stands in stark contrast to its pro-tumorigenic functions in established GBM, highlighting how the same epigenetic regulator can be co-opted to drive opposing biological outcomes in different cellular contexts [316]. Other JMJD members contribute to distinct hallmarks of GBM. JMJD1C, in contrast to its oncogenic role in leukemia, acts as a tumor suppressor in glioma by promoting anti-tumor M1 macrophage polarization through the miR-302a-METTL3-SOCS2 axis [317]. Furthermore, JMJD10, a demethylase for the non-histone target, controls proliferation by regulating cyclins and CDKs [318] and is essential for maintaining DNA replication and genomic stability [319]. The transcription elongation factor JMJD6 was also identified as a critical in vivo-specific dependency that promotes GBM cell survival in the TME [320].

In short, the JMJD family of demethylases exerts pivotal yet complex roles in malignancies like leukemia and glioma, characterized by remarkable context-dependent functions. A comprehensive overview of the JMJD proteins, including their expression patterns in cancer, targeted histone demethylation sites, and associated oncogenic signaling pathways, is summarized in Table 2. Individual members, such as JMJD1C, JMJD3, and UTX, can paradoxically act as either oncogenes or tumor suppressors across different cancer types, cellular subpopulations, and microenvironment. They orchestrate core oncogenic attributes, including therapy resistance, metabolic reprogramming, and immune evasion, through fine-tuning key pathways like MYC, p53, and Wnt/β-catenin. This intricate involvement positions JMJD proteins as promising therapeutic targets. However, their functional duality also presents a challenge, implying that successful targeting will require precise patient stratification and a deep understanding of the TME.

**Table 2:** Clinical significance and therapeutic targeting of JMJD family proteins in cancer

JMJD Protein	Subfamily	Demethylation site	High expression	Low expression	Signaling pathway	Inhibitor
JMJD1 (KDM3)	JMJD1A	H3K9me1/2, H4R3me1/2	PCa [71, 321], Primary BCa [139], CRC [174]	Metastatic BCa [143], GC [248]	AR [71], Hedgehog [116], ER [142], p53 [144], Wnt/β-catenin [174, 182]	Pan-KDM: JIB-04 [322]
	JMJD1B		BCa [150], HCC [223], ALL [280]		Wnt/β-catenin [150, 182]	
	JMJD1C		CRC [181]	GBM [317]		JDI-10 [286], JDI-16 [323]
JMJD2 (KDM4)	JMJD2A	H3K9me1/2/3, H3K36me2/3, H3K4me2	PCa [119, 120], BCa [137, 324, 325], HCC [224], GC [253], GBM [303]		AR [118], cGAS-STING [118], Hippo [119], p53 [183, 224], AKT-mTOR [303]	PKF118-310 [326], Compound 6p [327], IOX1 [328], Tetrazolylhydrazides [329]
	JMJD2B		PCa [119], Luminal/ER^+^ BCa [151, 153, 330], CRC [185–188, 190, 194, 331], GC [250, 251], GBM [305]	Primary BCa [156]	ER [152, 153], UPR [155], PI3K/AKT [157], Wnt/β-catenin [185], STAT3 [193, 194]	Pan-JMJD2: NCDM-32B [151], ML324 [228], TACH101 [332, 333], Myricetin [334]; JMJD2B: Compound 4 [335]
	JMJD2C		PCa [119], BCa [151, 161], HCC [225], GBM [307, 308]		Wnt/β-catenin [307], HIF-1α [160], p53 [308]	QC6352 [161, 254], SD70 [290, 336]
	JMJD2D		CRC [79, 196, 197], HCC [226, 227], AML [292]		Wnt/β-catenin [79, 226], HIF-1α [195], Hedgehog [197], Notch [226]	5-c-8HQ [79, 196, 197, 226], Compound 33a [337], 24 s [338]
JMJD3、UTX	JMJD3	H3K27me1/2/3	HCC [229], GC [257, 258]	CRC [198]	AR [123], Wnt/β-catenin [199], CXCL12/CXCR4 [311]	GSK-J4 [121, 124, 126, 258, 298–300, 339, 340]
	UTX		BCa [87, 137], PCa [134], CRC [201], GBM [315]	AML [294]	ER [87], AKT [201], TGF-β/SMAD [232], Wnt/β-catenin [259]	
JMJD4			CRC [204]		p53 [205]	
JMJD5 (KDM8)			CRC [206]		AR [94], HIF-1α [95]	
JMJD6		H4R3me2s	BCa [162, 163], CRC [207], HCC [233, 235, 236]		TGF-β [163], p53 [164, 207], ERK [234, 235]	SKLB325 [234], iJMJD6 [236, 341], WL12 [342]
JMJD10			BCa [166], CRC [209], HCC [244, 245], GC [54, 269], GBM [318]		AP-1 [54]	
JARID1 (KDM5)	JARID1A	H3K4me1/2/3	PCa [134], HCC [237, 238], GC [261, 262, 264]		ER [170], AKT [170, 237], Wnt/β-catenin [211]	cyclopenta[c]chromen derivative 1 [171], Rhodium(III) complex [172], CPI-455 [237], ryuvidine [343]
	JARID1B		PCa [133], CRC [212, 213], HCC [239, 240], GC [265, 267],		Wnt/β-catenin [212, 213]	Pan-JARID1: CPI-455 [344], KDOAM-25 [345]; JARID1B: AS8351 [241], TK-129 [346]
	JARID1C		CRC [215, 218]			
	JARID1D			PCa [135, 136], HCC [242]	AR [136]	
JARID2		H3K27me3	HCC [243]			

Abbreviations: *ALL* acute lymphoblastic leukemia, *AML* acute myeloid leukemia, *AP-1* activator protein 1, *AR* androgen receptor, *BCa* breast cancer, *CRC* colorectal cancer, *ER* estrogen receptor, *ERK* extracellular signal-regulated kinase, *GBM* glioblastoma, *GC* gastric cancer, *HCC* hepatocellular carcinoma, *HIF-1α* hypoxia-inducible factor 1-alpha, *PCa* prostate cancer, *STAT3* signal transducer and activator of transcription 3, *TGF-β* transforming growth factor-beta, *UPR* unfolded protein response

## Pathogenesis of JMJDs in non-malignant diseases

**Fig. 6:**
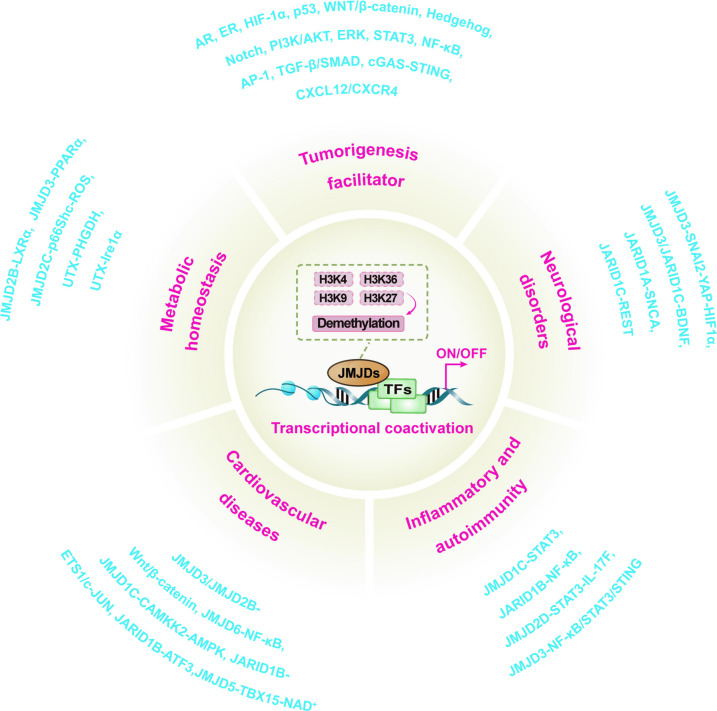
The pathogenic spectrum of JMJD family proteins. This pie chart illustrates the relative prevalence of human diseases linked to dysregulated JMJD functions (inner ring), which are mediated by the key signaling pathways regulated by JMJD proteins (outer ring). Disease segments include cancer, neurological disorders, inflammatory, autoimmune, cardiovascular, and metabolic diseases. Abbreviations: AR, androgen receptor; ATF3, activating transcription factor 3; BDNF, brain-derived neurotrophic factor; CAMKK2, calcium/calmodulin-dependent protein kinase; ER, estrogen receptor; HIF-1α, hypoxia-inducible factor-1 alpha; IL-17F, interleukin-17F; NF-κB, nuclear factor kappa-B; NAD^+^, nicotine adenine dinucleotide; PHGDH, phosphoglycerate dehydrogenase; PPARγ, peroxisome proliferator-activated receptor gamma; REST, RE-1 silencing transcription factor; SNCA, synuclein alpha; TBX15, T-box transcription factor 15; TFs, transcription factors

The regulatory functions of JMJD proteins extend far beyond carcinogenesis, playing critical roles in the pathogenesis of non-malignant diseases. Their capacity to interpret physiological and pathological signals and translate them into epigenetic changes positions them as key regulators across disparate disease contexts. This includes modulating processes central to neurodegeneration, tuning the response in inflammation and autoimmunity, and governing homeostasis in cardiovascular and metabolic systems. Dysregulation of these precisely regulated epigenetic mechanisms contributes significantly to disease initiation and progression. Here, we delineate the expanding roles of the JMJD family beyond oncology, reviewing their involvement in neurodegenerative, inflammatory, autoimmune, cardiovascular, and metabolic diseases (Fig. 6).

### JMJDs and neurological disorders

Neurological disorders encompass a spectrum of conditions including neurodegenerative diseases such as Alzheimer’s disease (AD), Huntington’s disease (HD), and Parkinson’s disease (PD); neurodevelopmental disorders like autism spectrum disorder (ASD) and intellectual disabilities; and complex psychiatric conditions such as depression. Members of the JMJD protein family critically influence the pathogenesis of these disorders by regulating neuronal development, synaptic plasticity, and the expression of key genes involved in cognitive function, often exhibiting context-dependent dual roles.

In AD, JMJD3 acts as a transcriptional activator for brain-derived neurotrophic factor (BDNF), a key protein for neuronal health and synaptic plasticity, by erasing the repressive histone mark H3K27me3 at its promoter [347]. Notably, the natural compound curcumin can upregulate JMJD3, thereby promoting BDNF expression and alleviating AD symptoms [348]. In contrast, JARID1C functions oppositely by demethylating the active mark H3K4me3 at the BDNF promoter, thereby suppressing its expression and exacerbating cognitive decline, Aβ deposition, and synaptic impairment [349]. Furthermore, JMJD2A was found to be highly expressed in a Drosophila AD model, where it exacerbates tau-induced neurodegeneration and motor deficits by disrupting heterochromatin structure [350]. Multi-omics analyses also suggest JMJD6 as a potential therapeutic target for AD [351]. In PD, JMJD3 demonstrates a protective role. It promotes the survival of dopaminergic neurons by activating the SNAI2-YAP-HIF1α signaling axis through H3K27me3 demethylation at the SNAI2 promoter [352]. Concurrently, JMJD3 promotes anti-inflammatory microglial polarization; its downregulation in PD shifts microglia toward a pro-inflammatory M1 phenotype, releasing factors like iNOS, IL-1β, and IL-6, which exacerbates neuroinflammation [353]. On the other hand, the accumulation of α-synuclein, a central event in PD, is associated with elevated H3K4me3 at the promoter of synuclein alpha (SNCA). CRISPR/dCas9-mediated targeted recruitment of the demethylase JARID1A to the SNCA promoter erases H3K4me3 and effectively reduces SNCA expression [354]. High-throughput genetic analyses have also identified JMJD10 as a potential risk gene for early-onset PD [355]. In HD, transcriptional repression of key neuronal genes such as *BDNF*, *PENK1*, and *DRD2* is associated with a marked loss of the active H3K4me3 mark at their promoters [356]. Inhibiting the H3K4 demethylase JARID1C restores H3K4me3 levels and has been shown to reverse disease symptoms, highlighting the therapeutic potential of targeting this epigenetic mechanism.

JMJD demethylases also constitute critical genetic determinants for neurodevelopmental disorders. In autism spectrum disorder (ASD), for instance, recurrent JARID1C mutations, including those causing a complete loss of enzymatic function and those with non-enzymatic effects, are strongly linked to disease susceptibility [357, 358]. A novel variant affecting the JARID1C 3’UTR-lncRNA has been linked to ASD with abnormal X chromosome inactivation [359]. Similarly, pathogenic variations in JARID1A are found in patients with language loss and intellectual disability [360]. Single-cell sequencing studies establish JARID1A as essential for the establishment and maintenance of hippocampal neuronal identity [361]. Its loss disrupts developmental pathways, culminating in premature maturation and abnormal neuronal morphology. In X chromosome-linked mental retardation (XLMR), JARID1C was identified as a causative gene. It functions as an H3K4me2/3 demethylase, and its inactivation disrupts RE-1 silencing transcription factor (REST)-mediated gene silencing, leading to spatiotemporal misexpression of neuronal genes [362–364]. The defect in its enzymatic activity directly correlates with the severity of patient symptoms. A unique peripheral blood DNA methylation signature in patients with JARID1C pathogenic variants offers a novel tool for molecular diagnosis [365]. Besides, mutations in JMJD1C are also implicated in other intellectual disabilities, including Rett syndrome [366]. In the complex pathogenesis of depression, JMJD proteins modulate the reward circuitry of brain. JMJD2D is downregulated in the nucleus accumbens during early stages of depression, while JMJD2A, JMJD2B, and JMJD2C are downregulated in later stages [367]. Inhibition of JMJD2 is sufficient to induce depressive-like behaviors in mice even without stress exposure [367]. Conversely, JMJD3 enhances depression susceptibility. In a maternal separation model, JMJD3 is upregulated in the prefrontal cortex and hippocampus, leading to reduced H3K27me3, microglial activation, and increased release of pro-inflammatory cytokines (TNF-α, IL-1β, IL-6). The JMJD3 inhibitor GSK-J4 reverses these pathological changes and alleviates depressive behaviors [368]. A cohort study also identified JMJD2C mutation as a significant risk factor in depression [369].

In summary, the JMJD family exerts profound and complex influences on neurological health and disease. Their functional duality often stems from the nature of the histone marks they remove, either repressive or activating, allowing them to fine-tune the transcriptional programs of key disease-related genes. Their recurring roles across disparate disorders highlight their fundamental importance in the nervous system and underscore their potential as therapeutic targets.

### JMJDs and inflammatory diseases

The JMJD family plays pivotal roles in regulating host responses to infection and tissue damage, fine-tuning the balance between pathogen clearance, inflammatory resolution, and tissue repair. In the context of viral and bacterial infections, JMJD proteins are critical regulators of viral latency and reactivation cycles. For Kaposi’s sarcoma-associated herpesvirus (KSHV), JMJD2A is targeted by the viral protein K-bZIP, which acts as a SUMO E3 ligase to modify JMJD2A at K471 [100, 370]. This SUMOylation acts as a molecular switch that activates viral gene transcription and replication, impacting the survival and angiogenesis of primary effusion lymphoma cells by regulating host genes like *IL-10* [371]. KSHV PAN RNA further recruits UTX and JMJD3 to activate lytic replication [372]. Similarly, for herpes simplex virus 1 (HSV1), inhibition of JMJD2, JMJD3, and UTX promotes the transition from latency to activation [373, 374]. For human immunodeficiency virus type 1 (HIV-1), the roles of JMJD proteins depend on their respective histone substrates. JARID1A/B and JMJD10 promote latency by erasing the active mark H3K4me3 and inhibiting transcription elongation, respectively [56, 375]. In contrast, UTX is necessary for reactivation by removing the repressive H3K27me3 mark [376]. JARID1C similarly promotes human papillomavirus (HPV) latency by erasing H3K4me3 [377], whereas JMJD2D interacts with the HBx protein of hepatitis B virus (HBV), stabilizing it and promoting viral transcription and replication [378].

During bacterial challenges, JMJD2D enhances host defense against intestinal pathogens by cooperating with STAT3 to demethylate H3K9me3 on the interleukin-17F (IL-17F) promoter, inducing antimicrobial peptide β-defensin production [379]. Conversely, pathogens like mycobacteria and *Salmonella* exploit JMJD3 to promote disease. Mycobacteria induce JMJD3 via TLR2 signaling, driving lipid metabolism genes (*e.g., Acsl1*, *Fat*) and M2 macrophage polarization to facilitate immune escape and foam cell formation [380]. *Salmonella* activates JMJD3 via its *Salmonella* pathogenicity island I (SPI1) effector, promoting M2 polarization and chronic infection through PPARδ [381]. Chronic *Salmonella* infection in gallstones also activates JMJD3, which upregulates oncogenes by removing H3K27me3, linking chronic inflammation to pre-cancerous lesions [382]. In contrast, JMJD3 plays a protective role in helminth infection by driving M2 macrophage polarization via demethylating the interferon regulatory factor 4 (IRF4) promoter [383].

In the hyperinflammatory state of sepsis, JMJD3 is upregulated in early-stage neutrophils, promoting mPR3 expression and subsequent TNFα/IL-1β release [384]. In later stages, UTX promotes the immunosuppressive function of MDSC by epigenetically activating the long non-coding RNA Hotairm1 [385, 386]. Mesenchymal stem cell-derived exosomal miR-27b can alleviate sepsis by targeting JMJD3 and dampening the NF-κB pathway [387]. Dexmedetomidine also attenuates sepsis-related inflammation and kidney injury by suppressing JARID1A and reducing H3K4me3 on NF-κB pathway genes [388]. Furthermore, the stability of JMJD is regulated during inflammation. For instance, TRIM14 prevents autophagic degradation of JMJD2D, thereby enhancing pro-inflammatory cytokine production [103].

Beyond infectious agents, JMJD members play context-dependent roles in tissue damage and repair, with their functions varying significantly across tissue types and disease states. Regarding metabolic and chronic damage, JMJD3 is a key regulator of chronic inflammation in diabetic wounds. It is activated via the IL-6-JAK-STAT3 pathway or by elevated palmitic acid through TLR4-MyD88 signaling, leading to the upregulation of the STING and NF-κB pathways and impaired healing [83, 389]. In keratinocytes, IL-1α induces JMJD3, which removes H3K27me3 from the NLRP3 promoter, promoting its transcription [390]. Paradoxically, JMJD3 also aids epithelial repair in keratinocytes by cooperating with NF-κB to activate Notch1, MMPs, and growth factor genes, thereby facilitating migration and wound closure [391, 392]. In acute organ injury models, JMJD3 exacerbates acute lung injury by promoting ferroptosis in alveolar epithelial cells via suppression of the Nrf2 pathway and by regulating adenosine A2A receptor (ADORA2A) to inhibit macrophage apoptosis and enhance inflammation [393, 394]. The JMJD3-C/EBPβ-JARID1A axis further amplifies lung inflammation by inhibiting IL4i1, shifting macrophages towards the M1 phenotype [395]. JMJD3 also promotes oxidative stress and inflammation in acute liver injury, while JMJD2A drives M1 polarization of liver macrophages and trichloroethylene-induced liver injury via the Wnt/β-catenin pathway [396, 397]. In contrast, JMJD3 exhibits protective effects in kidney injury, inhibiting renal tubular cell apoptosis, reducing STAT3-NF-κB-driven inflammation, and antagonizing pro-fibrotic TGF-β-SMAD3 signaling [398]. JMJD2C also protects against kidney injury by regulating autophagy and mitochondrial dynamics [399]. In central nervous system injury, UTX in vascular endothelial cells impedes spinal cord recovery by inhibiting vascular regeneration [400], whereas its deficiency promotes repair by enhancing neurogenesis and M2 macrophage polarization [401, 402]. Contrastingly, in neural stem cells, UTX activates the NF-κB pathway and inhibits migration inhibitory factor (MIF), exacerbating macrophage-mediated inflammation and impairing recovery [403]. JMJD3 contributes to the inflammatory response in endothelial cells after spinal cord injury by collaborating with NF-κB-C/EBPβ to activate IL-6 expression [404], and JMJD2A exacerbates cerebral ischemia–reperfusion injury by activating CXCL1 in astrocytes [405]. JARID1D has also been identified as a potential biomarker for traumatic brain injury [406].

### JMJDs and cardiovascular disease

The JMJD family of histone demethylases plays a critical and intricate epigenetic network that is fundamental to cardiovascular homeostasis and disease pathogenesis. Their functions, ranging from histone and RNA modification to direct protein hydroxylation, exhibit profound cell-type and context specificity, driving diverse outcomes in cardiac remodeling and vascular dysfunction.

In cardiac hypertrophy and remodeling, members of the JMJD family exhibit diverse functions. JMJD6 acts as a protective factor by demethylating p65 at R149, thereby inhibiting its nuclear translocation and pro-hypertrophic signaling [48]. Conversely, JMJD1C functions as a potent driver of pathology. It promotes cardiomyocyte hypertrophy through multiple mechanisms: it inhibits the calcium/calmodulin-dependent protein kinase (CAMKK2)-AMPK signaling axis [407], and it upregulates miR-200c-3p via H3K9me2 demethylation, leading to a cascade that suppresses PHF8 transcription [408]. Similarly, JMJD3 drives the hypertrophic gene program by specifically removing the repressive H3K27me3 mark from the β-MHC promoter [409]. This enzymatic function is conserved in cardiac fibrosis, where JMJD3 similarly removes H3K27me3 to activate β-catenin transcription in fibroblasts, promoting extracellular matrix deposition [410]. The fibrotic response is further exacerbated by JMJD2A, which epigenetically represses autophagy in fibroblasts by downregulating H3K9me3 at the Trim44 promoter, inducing a premature senescent, pro-fibrotic phenotype [411]. Complementing this, JARID1B drives pathological fibrosis by demethylating H3K4me3 at the promoter of the antifibrotic factor activating transcription factor 3 (ATF3), thereby unleashing TGF-β signaling [412].

Cardiomyocyte energy metabolism and survival are finely tuned by JMJDs, with disruptions leading to distinct cardiomyopathies. JMJD5 safeguards cardiac metabolic homeostasis by repressing T-box transcription factor 15 (TBX15) to maintain the nicotine adenine dinucleotide (NAD^+^) synthesis pathway, and its loss triggers a metabolic deficit leading to dilated cardiomyopathy [96]. A novel paradigm is illustrated by JMJD4, which protects against dilated cardiomyopathy not via demethylation but through hydroxylation of the metabolic enzyme PKM2, targeting it for chaperone-mediated autophagy and preventing its pathological accumulation, thereby ensuring efficient metabolic flux in the heart [42]. In acute injury, JMJD5 also protects cardiomyocytes from oxygen–glucose deprivation by upregulating the HIF-1α-BNIP3 axis to promote mitophagy [413].

During atherosclerosis progression, the roles of JMJD proteins are cell-type specific. In macrophages, JMJD1C aggravates lipid deposition and inflammation by mediating H3K9 demethylation to enhance proprotein convertase subtilisin/kexin type 9 (PCSK9) transcription [414]. Surprisingly, myeloid JMJD3 appears to play a complex, context-dependent role, with its deficiency leading to more advanced atherosclerosis, suggesting a role in modulating plaque stability [415]. This effect is potentially mediated by its control of a pro-fibrotic transcriptome in foam cells [416]. Within the endothelium, flow disturbance activates the mechanosensor Piezo1, which upregulates JARID1B via the transcription factors ETS-1 and c-JUN [417]. Elevated JARID1B then promotes endothelial inflammation by reducing H3K4me3 levels at the promoters of inflammatory genes, thereby accelerating atherosclerosis [417]. However, under ischemic conditions, JMJD2B promotes compensatory angiogenesis in diabetic limbs by activating the Wnt/β-catenin pathway [418], while JMJD8 fuels the angiogenic process by interacting with PKM2 to boost endothelial mitochondrial respiration [49]. In addition, JMJD6 distinctly regulates angiogenic sprouting by controlling the alternative splicing of VEGF receptor Flt1 mRNA, thereby modulating the production of its soluble inhibitory isoform to regulate endothelial responses [419].

The regulatory scope of JMJDs extends to vascular tone and neurovascular disease. In smooth muscle cells, a hypertension-associated genetic variant reduces JMJD3 expression, leading to an imbalance between endothelin receptors, characterized by decreased endothelin receptor type B (EDNRB) and increased endothelin receptor type A (EDNRA), and ultimately results in elevated blood pressure [420]. Furthermore, JMJD2A exacerbates ischemic brain injury by regulating microglial polarization through multiple axes, including MDM2-CTRP3 and SPINK5 [421, 422], while JMJD3 aggravates cerebrovascular lesions in cerebral amyloid angiopathy by epigenetically upregulating poly(ADP-ribose) polymerase 16 (PARP16) [423].

Importantly, the same protein or pathway can yield opposite outcomes based on cellular context. Both JMJD3 and JMJD2B activate Wnt/β-catenin, yet the former drives maladaptive fibrosis in fibroblasts [410] while the latter promotes adaptive angiogenesis in endothelium [418]. Similarly, JARID1B is pathogenic in stressed fibroblasts and endothelium [412, 417], but is essential for physiological angiogenesis by repressing the anti-angiogenic factor homeobox A5 (HOXA5) [424]. This profound context-dependence is further exemplified by KDM4A. While it acts as a potent driver of pathology in cardiac fibrosis and neuroinflammation [411, 421, 422], it is paradoxically recruited by the transcription factor ETV2 to serve as an indispensable co-activator for hematovascular lineage generation and vascular regeneration [425]. This contrast highlights that the functional outcome of a JMJD protein is not intrinsic but is dictated by the upstream signals and cellular milieu in which it operates.

Members of the JMJD family serve as pivotal, context-sensitive regulators of the cardiovascular epigenetic landscape. Their functions, which encompass modifying both histone and non-histone substrates, dictate critical gene networks underlying pathological hypertrophy, fibrosis, and angiogenesis. The ultimate biological outcome, whether protective or detrimental, is not inherent to the enzyme itself, but is precisely determined by the specific cellular milieu, disease state, and tissue microenvironment.

### JMJDs and autoimmune diseases

JMJD proteins are critically involved in the pathogenesis of autoimmune diseases by dynamically regulating the epigenetic landscape of immune and tissue-resident cells. In rheumatic diseases such as osteoarthritis and rheumatoid arthritis, JMJD3 emerges as a key pathogenic factor. Its expression is elevated in affected cartilage, and its inhibition alleviates inflammation and cartilage matrix degradation by blocking the NF-κB pathway [426]. Beyond this general inflammatory role, a pivotal mechanism in osteoarthritis involves JMJD3-driven pathological phenotypic switching. JMJD3 removes the repressive H3K27me3 mark from developmentally poised, bivalent genes in chondrocytes, leading to their aberrant activation and promoting a destructive hypertrophic, growth plate-like phenotype that is central to cartilage degradation [427]. Furthermore, abnormal mechanical stress induces JMJD3 in chondrocytes, which then activates the expression of pro-apoptotic factors like nuclear receptor subfamily 4 group A member 1 (NR4A1) by removing H3K27me3 from its promoter, leading to chondrocyte apoptosis and matrix degradation [428]. The function of UTX contrasts with JMJD3 in the articular setting. UTX deficiency in cartilage inhibits the expression of PRC2 core components Eed and Suz12, reducing H3K27me3 enrichment at promoters of key genes like the anabolic master regulator *Sox9* and the growth factor *Igf2*, thereby derepressing these cartilage-protective genes and promoting matrix synthesis [429]. This indicates that while both demethylate H3K27me3, their target genes and effects on cartilage homeostasis are distinct and often opposing.

In rheumatoid arthritis, the pathogenic role of JMJD3 extends to the synovium. H₂S, produced by cystathionine γ-lyase (CSE), negatively regulates JMJD3, suppressing the expression of inflammatory genes like *TLR2* to mitigate arthritis [430]. Conversely, elevated JMJD3 regulates the proliferation and migration of fibroblast-like synoviocytes (FLS), a key driver of joint pathology [431]. This effect is mediated through its removal of the repressive H3K27me3 from the promoter of proliferating cell nuclear antigen (PCNA), thereby unleashing the hyperproliferative and invasive capacity of FLS [431]. Other JMJD members contribute significantly to arthritis pathogenesis. In macrophages within a collagen-induced arthritis model, JARID1B promotes NF-κB activation and pro-inflammatory cytokine production by repressing the *Nfkbia* gene, which encodes the NF-κB inhibitor IκBα [432]. JMJD1C, however, limits plasma cell overactivation and alleviates arthritis by demethylating and inhibiting the phosphorylation and activity of STAT3 protein [433].

In systemic lupus erythematosus (SLE), the roles of JMJD proteins exhibit greater complexity and context-dependency. JMJD3 can play a protective role in CD4^+^ T cells by binding to the hematopoietic progenitor kinase 1 (HPK1) promoter and removing H3K27me3, thereby epigenetically activating HPK1 to restrain excessive T cell activation and autoimmunity [434]. In contrast, increased activity of both JMJD3 and UTX in SLE monocytes, fueled by immunometabolic reprogramming and elevated 2-OG production, drives pathogenesis by removing H3K27me3 from interferon-stimulated gene promoters [435]. This establishes a trained immunity phenotype, resulting in the persistent hyperactivation of the type I interferon pathway, a hallmark of SLE [435]. Further complexity in SLE involves other JMJD family members. In B cells, JARID1A is significantly increased and promotes activation by downregulating the critical immune checkpoint protein tumor necrosis factor alpha-induced protein 3 (TNFAIP3, also known as A20) [436]. It achieves this by decreasing the activating H3K4me3 mark at the A20 promoter, facilitating B cell hyper-reactivity [436]. Clinical studies further identify JMJD10 as a potential biomarker in SLE, with significantly elevated serum levels and gene expression that correlate with disease activity and severity [437].

Beyond arthritis and SLE, JMJD proteins are implicated in other autoimmune conditions. In ulcerative colitis, JMJD3-mediated removal of H3K27me3 is crucial for skewing CD4^+^ T cell differentiation towards the pro-inflammatory Th17 lineage and away from the regulatory Treg lineage, thereby disrupting mucosal immune homeostasis [438]. In systemic vasculitis, cross-phenotype genetic analyses have identified JMJD2C as a novel shared risk locus, linking epigenetic dysregulation to this group of vascular inflammatory diseases [439].

Collectively, the studies presented here firmly establish the JMJD family as a class of promising therapeutic targets for inflammatory and autoimmune diseases. They integrate signals from pathogens, metabolites, and cytokines to orchestrate gene expression programs in both immune and tissue-resident cells. Their actions, whether protective or pathogenic, are exquisitely context-dependent, determined by cell type, disease microenvironment, and interacting protein partners.

### The role of JMJDs in other diseases

JMJD demethylases also function as master regulators of systemic metabolic homeostasis, governing fundamental processes such as lipid flux, energy expenditure, and autophagic function across multiple organs [440–442]. This systemic metabolic control implicates them directly in the pathogenesis of obesity, non-alcoholic fatty liver disease (NAFLD), and diabetes. In the liver, the demethylase JMJD2B can promote the expression of LXRα-dependent lipogenic genes by erasing H3K9me2/3 marks, potentially driving hepatic steatosis [443]. Conversely, hepatic JMJD3 activity is induced during fasting by FGF21-PKA signaling, which phosphorylates JMJD3 at Thr-1044, facilitating its nuclear translocation and partnership with PPARα to co-activate genes for hepatic autophagy and lipid degradation [104]. In adipose tissue, JMJD1A serves as a central regulator of thermogenesis and energy expenditure. Its function is regulated by distinct post-translational modifications in response to specific stimuli. Under acute cold stress, β-adrenergic receptor-activated PKA phosphorylates JMJD1A at S265, facilitating the assembly of a transcriptional complex with PPARγ and the SWI/SNF chromatin remodeler to rapidly induce thermogenic genes like *Ucp1* [72, 73]; a similar phosphorylation-dependent, demethylase-independent mechanism mediates the response to heat stress via mitogen- and stress-activated protein kinase 1 (MSK1) [444]. During chronic cold adaptation, JMJD1A’s demethylase activity takes precedence, stabilizing the expression of beige fat-specific genes (*e.g., Ucp1*, *Cidea*) by erasing H3K9me2 marks, thereby promoting adipose tissue browning and mitochondrial biogenesis [445]. The physiological importance of these mechanisms is underscored by the phenotype of JMJD1A^S265A^ mutant mice, which develop insulin resistance under a high-fat diet and cold stress, and by the observation that disruption of its H3K9 demethylase activity (*e.g.,* at the Pgc1a/b enhancer) impairs energy expenditure, exacerbates obesity and metabolic disorders [445, 446]. Complementing this, JMJD2B enhances lipolysis and oxidative metabolism by activating genes for fatty acid oxidation and mitochondrial function through H3K9me3 demethylation [447]. Meanwhile, within adipose tissue macrophages, the demethylase UTX inhibits M2 polarization and suppresses adipose thermogenesis by reducing H3K27me3 at the *Ire1α* locus, thereby promoting obesity [448]. The JMJD family also orchestrates metabolic communication between organs. A striking example is found in the kidney, where ubiquitination-mediated degradation of UTX inhibits serine synthesis by downregulating PHGDH, leading to hyposerinemia that subsequently promotes lipid accumulation in both the kidney and liver [449]. Furthermore, in the vascular endothelium, a key interface between metabolic and cardiovascular systems, endothelial-specific knockdown of JMJD2C in obese mice improves endothelium-dependent vasodilation by reducing p66Shc expression and ROS production [450]. Thus, JMJD demethylases function as central nodes in an inter-organ epigenetic network that governs systemic metabolic homeostasis.

## Therapeutic targeting of JMJD demethylases

The discovery of JMJD protein family has not only unveiled a crucial layer of epigenetic regulation but also presented a novel class of promising therapeutic targets for a wide spectrum of diseases. As detailed previously, by modulating key signaling pathways (*e.g.,* AR, Wnt/β-catenin, HIF, NF-κB, and STAT3) and downstream genes (*e.g., MYC*, *CCND1*, *SNAI2*, *BDNF*, *PCSK9*, *UCP1*), JMJD dysregulation is mechanistically implicated in the pathogenesis of a wide spectrum of diseases, including cancer, neurodegenerative, inflammatory, autoimmune, cardiovascular, and metabolic disorders [30, 79, 160, 196, 349, 352, 389, 414, 445]. This established causality provides a strong rationale for therapeutic intervention. The therapeutic potential of JMJD proteins is further supported by the development of selective small-molecule inhibitors targeting various JMJD family members (Table 2). Translating this knowledge into clinical strategies, however, requires navigating the complexities of isoform selectivity, functional redundancy, and context-dependent roles. This section outlines the development of these inhibitors, explores complementary strategies targeting their regulatory networks and effector pathways, discusses rational combination therapies, and analyzes the ongoing challenges and future directions in the field.

### Targeting JMJDs through small-molecule inhibitors

The development of JMJD small-molecule inhibitors represents an evolutionary journey from breadth to precision. Early efforts primarily relied on the establishment of miniaturized high-throughput screening platforms [451] or structure-based rational design centered on the known co-substrate 2-OG [452], yielding foundational pioneering compounds. These early molecules, such as GSK-J4 (targeting KDM6 family) and the pan-KDM inhibitor JIB-04 [322, 453], though possessing limited selectivity, served as powerful chemical probes that significantly advanced our understanding of JMJD biology. Notably, GSK-J4 demonstrated encouraging anti-cancer activity across various tumor models. GSK-J4 was shown to effectively suppress the expansion and self-renewal capacity of BCa stem cells [454], offering a novel strategy for preventing tumor relapse. This approach of targeting CSCs is further validated by the recent development of zavondemstat (TACH101), a pan-JMJD2 inhibitor, which was also observed to reduce the population of tumor-initiating cells following treatment [332]. The translational potential of this strategy is supported by an initial clinical study of TACH101 in heavily pre-treated patients with advanced solid tumors. The compound was very well tolerated with no dose-limiting toxicities observed, and it demonstrated encouraging preliminary efficacy, with stable disease achieved in 44% of evaluable patients, including durable clinical benefit (≥ 6 months) in patients with advanced solid tumors [333]. Critically, the therapeutic potential of GSK-J4 extends to CRC, where it was shown to potently eradicate tumor-initiating cells and suppress stemness-associated gene signatures by inducing global enhancer reprogramming, thereby inhibiting tumor growth and sensitizing CRC cells to chemotherapy [339]. GSK-J4 was also demonstrated to effectively reverse the oncogenic effects driven by JMJD3 overexpression, highlighting its potential as a therapeutic option for GC [258]. Besides, in AML, it not only induced cell cycle arrest and apoptosis via endoplasmic reticulum stress [455] but also triggered degradation of the transcription factor CREB through a PKA and proteasome-dependent mechanism [456]. Concurrently, studies revealed significant synergistic effects between GSK-J4 and conventional chemotherapeutic agents. For instance, in KRAS-mutant anaplastic thyroid cancer, the combination of GSK-J4 and doxorubicin achieved significant tumor suppression at low doses [457]; similarly, in AML models, GSK-J4 combined with decitabine demonstrated synergistic anti-leukemic effects [455].

However, researchers recognized that high selectivity is crucial for achieving genuine therapeutic value and reducing off-target toxicity. A major challenge in targeting JMJD1A lies in the structural and functional redundancy across the JMJD family. The catalytic JmjC domain is highly conserved, raising the risk that inhibitors may inadvertently affect JMJD1A paralogs or even members with opposing functions in different cancer contexts. To this end, they have focused their efforts on “precise targeting”. Guided by structural biology insights, researchers resolved the complex structures of several JMJD family members bound to inhibitors. For example, the structural elucidation of the JMJD2B/2,4-pyridinedicarboxylic acid/H3K9me3 ternary complex [335], and the analysis of JARID1B binding modes with three distinct inhibitors [60], provided a foundation for the rational design of highly selective inhibitors. Based on these structural insights, computer-aided virtual screening and fragment-based drug design were widely employed. Researchers not only discovered novel inhibitor scaffolds, such as pyrazolo[1,5-a]pyrimidine-3-carbonitrile [458] and 4,6-diarylquinoxaline [337], but also optimized lead compounds. This period yielded numerous excellent candidate molecules: the pyrazole-based derivative TK-129 exhibited inhibition activity against JARID1B at 44 nM and demonstrated favorable oral bioavailability (42.37%) and therapeutic efficacy in a myocardial fibrosis model [346]; compound 24 s, based on a 2-(aryl(pyrrolidine-1-yl)methyl)phenol scaffold, showed over 1500-fold selectivity for towards JMJD2D versus JMJD2A as well as other JMJD subfamily members and possessed a unique non-2-OG competitive inhibition mechanism [338].

Particularly noteworthy is the ongoing expansion of therapeutic concepts for these inhibitors. The natural product myricetin was identified as a pan-KDM4 inhibitor, and researchers successfully overcame its poor bioavailability using poly lactic-co-glycolic acid (PLGA) encapsulation, demonstrating significant efficacy in PCa models when combined with enzalutamide [334]. The emergence of the first JMJD3/HDAC dual inhibitor represents a novel direction in epigenetic drug development [459]. Complementing these approaches, the proteolysis-targeting chimera (PROTAC) technology offers a paradigm-shifting strategy. Unlike inhibitors that merely block enzymatic activity, PROTAC are bifunctional molecules designed to recruit the target protein (*e.g.,* a JMJD family member) to an E3 ubiquitin ligase, leading to its ubiquitination and subsequent proteasomal degradation [460]. This event-driven mechanism can not only achieve potent and durable effects but also potentially overcome issues of functional redundancy and catalytic site inhibitor resistance by removing the entire protein scaffold.

### Targeting JMJDs via upstream regulatory networks

While direct catalytic inhibition is a primary strategy, the complex biology of JMJD demethylases necessitates complementary approaches. Targeting the upstream regulatory layers that control JMJD activity presents an alternative avenue for therapeutic intervention. JMJD protein function is orchestrated by upstream regulatory and post-translational inputs, presenting viable indirect therapeutic levers. A key regulatory layer is cellular metabolism, as intracellular levels of the cofactor 2-OG and its antagonist succinate directly modulate JMJD demethylase activity [81]. Inhibiting 2-OG-producing enzymes (*e.g.,* IDH1/2) or supplementing succinate can indirectly suppress JMJD function, particularly in cancers with dysregulated metabolism [461]. Another crucial layer is governed by PTMs, which precisely regulate JMJD protein stability, localization, and activity. For instance, phosphorylation of JMJD1A at S265 by PKA is essential for its role in adipose thermogenesis [72, 73], while its phosphorylation by JAK2 promotes STAT3 signaling in CRC [102]. Furthermore, the SUMOylation of JMJD2A by the KSHV protein K-bZIP activates viral replication [100, 370]. Collectively, these mechanisms highlight that targeting the upstream enzymes, such as kinases, acetyltransferases (*e.g.,* p300), or ubiquitin ligases (*e.g.,* HUWE1, Fbxo22), offers a viable strategy for the indirect and context-specific modulation of JMJD protein function [97, 99]. This strategy is exemplified therapeutically by using the HSP90 inhibitor 17-AAG to induce degradation of JARID1B and reverse chemotherapy resistance in GC [266].

### Targeting JMJDs with rational combination therapies

Because JMJD demethylases occupy central positions in transcriptional regulation, DNA repair, metabolic adaptation, and immune modulation, combining JMJD-targeted interventions with other therapeutic modalities offers a compelling strategy to enhance efficacy and overcome resistance. One major avenue is the concurrent suppression of core oncogenic pathways where JMJD proteins serve as critical epigenetic co-factors. A prime example is the JMJD1A-c-Myc axis: JMJD1A drives prostate and other cancers by both transcriptionally activating and post-translationally stabilizing c-Myc [71]. Thus, combining JMJD1A inhibition with direct c-Myc inhibitors (*e.g.,* Omomyc-based peptides) or agents targeting Myc-dependent transcription and metabolism could yield synergistic effects [462]. In hormone-driven cancers, JMJD members (*e.g.,* JMJD1A, JMJD2, KDM6) function as key epigenetic co-activators for nuclear receptors like AR and ER suggests that integrating JMJD inhibitors with AR/ER antagonists (*e.g.,* enzalutamide, fulvestrant) or degraders (*e.g.,* PROTACs) could effectively overcome resistance mechanisms in this collaborative signaling [87, 118, 142]. Similarly, in hypoxic tumors, simultaneously targeting the JMJD2C-HIF-1α or JMJD5-PKM2-HIF-1α complexes and their metabolic effector proteins (*e.g.,* HIF-1α, PKM2) may decrease tumor survival more effectively than single-agent therapy [95, 160].

A parallel strategy involves combining JMJD inhibitors with agents targeting complementary epigenetic regulators. This is evident in the antagonistic relationship between the H3K27 methyltransferase EZH2 and the demethylases JMJD3/UTX, which oppositely regulate cellular differentiation and plasticity. Studies in liver and pancreatic differentiation models show that inhibiting JMJD3/UTX sustains a stem-like state through maintained H3K27me3, while EZH2 inhibition promotes differentiation by reducing this repressive mark [463, 464]. Such dynamics offer a rationale for cancer treatment: concurrent inhibition of both JMJD3/UTX and EZH2 can suppress the reversible turnover of H3K27me3, resulting in durable silencing of oncogenes across various tumors. Similarly, in acute leukemias, synergistic killing is achieved by combining an inhibitor of the H3K9 demethylase KDM4C with an agent that disrupts the MLL methyltransferase complex [336]. The development of a JMJD3/HDAC dual inhibitor further demonstrates the potential of simultaneously targeting multiple epigenetic nodes [459].

The intimate involvement of JMJD proteins in the DDR establishes a strong rationale for combining their inhibitors with genotoxic therapies. JMJD1A promotes double-strand break repair via c-Myc and p300 recruitment [99], while JARID1B facilitates DDR in GC [266]. Pharmacological inhibition of these demethylases is therefore predicted to sensitize tumors to PARP inhibitors, radiation, and DNA-damaging chemotherapies. This synthetic-lethal framework is further validated by studies showing synergy between the KDM6 inhibitor GSK-J4 and agents like decitabine or doxorubicin [455, 457]. Besides, the capacity of JMJD proteins to sculpt the tumor immune microenvironment opens avenues for combination with immunotherapy. JMJD2B- and JMJD2D-mediated regulation of PD-L1 and other immune checkpoints indicates that JMJD inhibition may convert poorly immunogenic tumors into immune-responsive states, thereby improving the effectiveness of immune checkpoint blockade [189, 196].

Collectively, the strategic integration of JMJD-targeted agents with pathway inhibitors, other epigenetic drugs, standard cytotoxic/radiation therapies, and immunomodulators enables a multi-layered attack on cancer. These rational combinations, grounded in the intricate biology of JMJD networks, hold significant promise for achieving more durable clinical responses.

### Challenges and novel strategies in JMJD-targeted therapy

Despite the promising outlook, the development path for JMJD-targeted therapies remains fraught with challenges. The primary hurdle is the issue of selectivity arising from high sequence homology within JMJD families. While successful cases exist for targeting subfamilies like JMJD2 or JARID1 [344, 465], achieving absolute selectivity across subfamilies or for specific isoforms remains a significant challenge. Secondly, validating the JMJD family as therapeutic targets poses a significant challenge. Functional redundancy among many JMJD members and their potentially context-dependent roles add layers of complexity. Research in PCa revealed that the efficacy of the JMJD3 inhibitor GSK-J4 strictly depended on AR status, inhibiting growth in androgen-dependent tumors while promoting it in androgen-independent models [123]. This complexity underscores the necessity for precise target validation within specific disease contexts and molecular subtypes. Furthermore, in vivo efficacy and delivery are critical for translational success. Although JIB-04 demonstrated good oral bioavailability (44.4%) in rats [466], ensuring effective penetration of physiological barriers like the blood–brain barrier remains challenging. The finding that JIB-04 can cross the blood–brain barrier and prolong survival in a glioblastoma model offers hope for treating neurological disorders [467].

Confronting these challenges, the field is cultivating a series of breakthrough strategies: (1) Allosteric inhibition and novel mechanisms of action are current research hotspots. While most existing inhibitors competitively occupy the 2-OG binding site, recent studies revealed that JIB-04 exerts its effect by disrupting oxygen binding in the active site, a unique mechanism accompanied by significant protein conformational changes [468]. Meanwhile, systematic mapping studies of the JMJD2D histone-binding pocket [469] provide a structural basis for developing allosteric inhibitors targeting this region. (2) Innovative delivery strategies and drug repurposing show considerable potential. To address the bioavailability issues of the natural product myricetin, researchers employed Food and Drug Administration (FDA)-approved PLGA material for encapsulation, significantly enhancing its in vivo anti-tumor efficacy [334]. Drug repurposing strategies have also made important strides, with known Wnt pathway inhibitor PKF118-310 and the compound ryuvidine being re-identified as JMJD2A and JARID1A inhibitors, respectively [326, 343], substantially accelerating the drug discovery process. (3) Synergistic combination therapies are becoming a mainstay. In MLL-rearranged acute leukemia, the combination of the JMJD2C inhibitor SD70 and the menin inhibitor MI-503 produced potent anti-leukemic effects through synergistic downregulation of MYC target genes [336]. In diffuse intrinsic pontine glioma (DIPG), combining GSK-J4 with the mutant p53-targeting agent APR-246 significantly enhanced the efficacy of radiotherapy [470]. As an illustrative example at the individual member level, JMJD1A inhibition may also provide strong synergistic opportunities, given that JMJD1A integrates metabolic cues, hypoxic signaling, and DNA damage responses, and its activity is regulated by 2-OG/succinate levels with depletion capable of activating the cGAS-STING pathway and remodeling the tumor immune microenvironment; therefore, inhibiting JMJD1A may synergize with DNA-damage-based therapies, metabolic interventions, and immune checkpoint blockade to achieve enhanced therapeutic efficacy. (4) The exploration of novel molecular modalities is particularly revolutionary. The recent advent of a peptide inhibitor selectively targeting JARID1C marks a paradigm shift in the field [471]. Not only does this peptide inhibitor demonstrate exceptional subtype selectivity, but it also significantly suppresses tumor growth in vivo, offering a novel strategy to overcome the selectivity bottleneck faced by small-molecule inhibitors. (5) Another groundbreaking strategy involves targeted protein degradation, particularly through PROTAC technology, which represents a highly promising avenue to address the dual challenges of selectivity and functional redundancy. By designing PROTACs that bind to non-catalytic, subtype-specific regions of a JMJD protein, it is possible to achieve degradation selectivity even among highly conserved family members. Preliminary successes in degrading other epigenetic targets have sparked efforts to develop JMJD-targeting PROTACs [460]. These degraders hold the potential to not only inhibit enzymatic function but also disrupt the non-catalytic scaffolding roles of these proteins, offering a more comprehensive therapeutic effect.

The therapeutic landscape of JMJD demethylases reflects the broader evolution of epigenetic drug development, from early non-selective probes to highly refined inhibitors, protein degraders, and biomarker-guided combination strategies. JMJD proteins function as central nodes in oncogenic, metabolic, and immune regulatory networks; thus, their effective targeting requires a multi-dimensional approach that integrates direct enzymatic inhibition, upstream regulatory modulation, and rational combinatorial therapies. As advances in structural biology, chemical biology, and precision oncology converge, JMJD-targeted therapeutics are poised to transition from conceptual promise to clinical reality.

## Conclusions

The JMJD protein family functions as pivotal dynamic epigenetic editors, critically governing gene expression programs in a wide spectrum of pathologies, including cancer, neurodegeneration, inflammation/autoimmunity, and metabolic homeostasis. These enzymes, which precisely erase methyl marks from histone (*e.g.,* H3K9, H3K27, H3K4) and non-histone substrates in an 2-OG- and Fe^2^⁺-dependent manner, serve as central hubs linking intracellular metabolic status and extracellular signals to the transcriptional machinery [15, 21, 22]. The established oncogenic roles of members such as JMJD1A, JMJD2C, and UTX have fueled the development of small-molecule inhibitors like GSK-J4 and JIB-04, which show considerable promise in preclinical models [121, 127, 466]. However, the journey from these promising preclinical findings to effective clinical therapies is fraught with challenges, primarily due to the complex, context-dependent roles of JMJD members, their intricate interplay with cellular metabolism and hypoxia signaling, and the current limitations in achieving selective pharmacological inhibition.

A major obstacle to precisely targeting JMJD proteins is their pronounced context-dependent functionality and potential functional redundancy. We emphasize that functional redundancy represents a defining characteristic of the JMJD protein family, wherein individual members can exert opposing biological roles across different diseases, or even within distinct cell types or stages of the same pathology. For instance, UTX acts as a tumor suppressor in GC and AML [86, 259] yet serves as an oncogenic co-activator in CRPC and specific T-ALL subtypes [121, 296]. Similarly, JMJD3 protects dopaminergic neurons in PD [352] but drives malignancy in numerous cancer types [126, 229]. These context-specific functions arise from the multi-layered regulation of JMJD proteins. Firstly, JMJD expression is tightly controlled by upstream transcription factors and signaling pathways. For example, JMJD2B is transcriptionally activated by ERα and HIF-1α [152, 154], coupling its expression to hormonal and hypoxic cues. Secondly, PTMs, including phosphorylation, ubiquitination, and SUMOylation, dynamically regulate the catalytic activity, subcellular localization, and substrate specificity of these enzymes [100, 101, 370]. Thirdly, the enzymatic activity of JMJD proteins is crucially dependent on the cellular metabolic state, as it is directly regulated by the concentration of key metabolites, including the essential co-substrate 2-OG and its competitive inhibitors succinate and fumarate [15, 81]. However, the molecular determinants that govern these functional switches remain poorly defined, and the extent to which distinct JMJD members compensate for one another to maintain epigenetic homeostasis is largely unexplored. Addressing these gaps will require conditional knockout models, single-cell multi-omics platforms (*e.g.,* scRNA-seq combined with scATAC-seq), and advanced proteomic strategies to delineate the context-specific functional landscape of JMJD proteins. Such knowledge will be critical for patient stratification and the development of precise therapeutic interventions.

JMJD protein family serves as intrinsic sensors of cellular metabolic status, with their catalytic activity directly governed by the intracellular 2-OG/succinate ratio and hypoxic signaling. This unique attribute positions them as master integrators of metabolic cues, the HIF pathway, and chromatin dynamics. The functional integration between JMJD proteins and HIF signaling is exemplified by the direct transcriptional upregulation of JMJD members, including JMJD1A, JMJD2B, and JMJD2C, by stabilized HIF-1α under hypoxia [154, 160, 221]. Paradoxically, the concomitant metabolic reprogramming and accumulation of succinate can concurrently inhibit their enzymatic activity [15]. This concurrent upregulation and potential inhibition may form a sophisticated feedback loop, fine-tuning JMJD activity and potentially redirecting it to specific genomic loci or non-histone substrates. Critically, JMJD proteins and HIF often form functional complexes on chromatin, as demonstrated by JMJD2C, which is recruited to HIF response elements to co-activate glycolysis-related genes like *LDHA* and *PDK1* by removing the repressive H3K9me3 mark, thereby amplifying the Warburg effect [160]. This epigenetic regulation is functionally coupled to chromatin remodeling. The demethylation reactions catalyzed by JMJD are essential for chromatin remodeling complexes. For instance, JMJD2B-mediated erasure of H3K9me3 creates a permissive state that facilitates the recruitment of the SWI/SNF complex, which subsequently remodels nucleosomes to grant the transcription machinery access to promoters [153]. In a parallel cooperative mechanism, UTX collaborates with histone acetyltransferases like p300/CBP to sculpt active enhancer landscapes, simultaneously erasing repressive H3K27me3 while depositing activating H3K27ac [87]. The cumulative evidence positions JMJD proteins at the nexus of a centralized “metabolism-epigenetics-chromatin remodeling” axis, underscoring the imperative to decode its molecular basis in future research. To this end, investigations should leverage proteomics and chromatin conformation capture techniques to map the interactome and genomic localization of specific JMJD proteins under various metabolic stresses.

Beyond intrinsic oncogenic roles, JMJD proteins critically shape the TME to foster immune evasion, thereby presenting a highly promising therapeutic frontier. Emerging evidence elucidates diverse mechanisms of JMJD-mediated immunosuppression. For instance, JMJD2A suppresses the innate cGAS-STING pathway [118], while JMJD2D promotes immune evasion by directly upregulating PD-L1 [196]. In contrast, in myeloid cells, JMJD3 drives M2 macrophage polarization [383], while the loss of its homolog UTX reprograms metabolism to expand MDSC [203], thereby suppressing anti-tumor immunity. Critically, therapeutic targeting of either JMJD2D or JMJD3 presents a viable strategy to enhance the efficacy of anti-PD-1 antibody treatment [196, 313]. Despite these insights, the current understanding remains fragmented. It remains unclear how JMJD proteins control specific immune cells, such as exhausted T cells, Tregs, and neutrophils. Furthermore, it is crucial to determine whether targeting JMJD in tumor cells, specific immune populations, or both, can effectively reverse the immunosuppressive TME and synergize with immune checkpoint blockade. A comprehensive dissection of JMJD functions within the “tumor-immune” circuit will require the combined use of immune cell-specific knockout models and humanized mouse models. The insights gained from such approaches will directly guide the development of novel and effective combination strategies based on epigenetic-immunotherapy.

Building on the promise of such combination immunotherapies, the clinical translation of JMJD inhibitors must first overcome the pervasive challenges of limited selectivity and inefficient in vivo delivery. The high conservation of the catalytic JmjC domain across the family complicates the design of highly selective inhibitors. Furthermore, many lead compounds exhibit suboptimal pharmacokinetic properties, including poor oral bioavailability and limited blood–brain barrier penetration. To realize the therapeutic potential of targeting JMJD proteins, future efforts should converge on a multi-pronged strategy. This includes the structure-guided design of allosteric inhibitors targeting subtype-specific pockets; the development of degradation-based modalities like PROTACs; the implementation of advanced delivery systems, such as nanoparticles, to improve pharmacokinetics; the exploration of synergistic drug combinations; and the expansion of drug repurposing screens to identify novel JMJD-inhibitory chemistries.

In summary, the JMJD protein family constitutes a sophisticated epigenetic regulatory network that critically influences development, homeostasis, and a spectrum of human diseases. Its members do not operate in isolation but are embedded in a web of metabolic sensing, signaling pathways, and chromatin remodeling complexes. Therefore, advancing the field necessitates the adoption of integrated approaches integrated approaches to systematically delineate their context-dependent functions, their central role in the metabolism-immune axis, and the principles governing their functional specificity. By solving these fundamental questions and overcoming the associated pharmacological hurdles, we will be poised to unlock novel precision therapeutic strategies targeting this crucial family of epigenetic regulators.

## Data Availability

Not applicable.

## Abbreviations

ABCC1: ATP-binding cassette subfamily C member 1
ACK1: Activated Cdc42-associated kinase 1
AD: Alzheimer’s disease
ADM: Adrenomedullin
ADORA2A: Adenosine A2A receptor
AGT: Angiotensinogen
AKT1: Protein kinase B
ALDH1A3: Aldehyde dehydrogenase 1 family member A3
ALL: Acute lymphoblastic leukemia
ALT: Alternative lengthening of telomeres
AML: Acute myeloid leukemia
APL: Acute promyelocytic leukemia
AR: Androgen receptor
ARID: AT-rich interaction domain
AR-V7: Androgen receptor splice variant 7
ASD: Autism spectrum disorder
ATF2: Activating transcription factor 2
ATF3: Activating transcription factor 3
ATRX: Alpha thalassemia/mental retardation syndrome X-linked
AXIN1: Axis inhibition protein 1
BCa: Breast cancer
BDNF: Brain-derived neurotrophic factor
BMP9: Bone morphogenetic protein 9
BRD4: Bromodomain-containing protein 4
C2HC4: Cys_2_-His-Cys_4_
CAMKK2: Calcium/calmodulin-dependent protein kinase
CCDC8: Coiled-coil domain containing 8
CDC6: Cell division cycle 6
CDK4: Cyclin-dependent kinase 4
CML: Chronic myeloid leukemia
CMML: Chronic myelomonocytic leukemia
COP1: Constitutive photomorphogenic 1
COX-2: Cyclooxygenase-2
CBP: CREB-binding protein
CRC: Colorectal cancer
CREB: CAMP response element-binding protein
CRPC: Castration-resistant prostate cancer
CSCs: Cancer stem cells
CSE: Cystathionine γ-lyase
CUL4B: Cullin 4B
DCS: Docetaxel, cisplatin, and S-1
DDB1: DNA damage-binding protein 1
DDR: DNA damage response
DIPG: Diffuse intrinsic pontine glioma
DSBH: Double-stranded β-helix
eIF2α: Eukaryotic translation initiation factor 2 alpha
eRF1: Eukaryotic release factor 1
E2F1: E2F transcription factor 1
EDNRA: Endothelin receptor type A
EDNRB: Endothelin receptor type B
EMT: Epithelial-mesenchymal transition
EpCAM: Epithelial cell adhesion molecule
ER: Estrogen receptor
ERE: Estrogen response element
ERG1: ETS-related gene 1
ERK: Extracellular signal-regulated kinase
ER^+^: ER-positive
ETBF: Enterotoxigenic *Bacteroides fragilis*
ETS-1: E26 transformation-specific 1
ETV1: ETS variant 1
Fbxo22: F-box protein 22
FBXW7: F-box/WD repeat-containing protein 7
FDA: Food and Drug Administration
FGFR4: Fibroblast growth factor receptor 4
FKBP4: FK506-binding protein 4
FLS: Fibroblast-like synoviocytes
FXYD3: FXYD domain-containing ion transport regulator 3
GABARAP: GABA type A receptor-associated protein
GATAL: GATA-like
GBM: Glioblastoma
GC: Gastric cancer
GDF15: Growth differentiation factor 15
GIST: Gastrointestinal stromal tumors
GSCs: Glioblastoma stem cells
H2A.X: Histone H2A variant X
H3K4: Histone H3 lysine 4
H3K9: Histone H3 lysine 9
H3K27: Histone H3 lysine 27
H3K36: Histone H3 lysine 36
H4R3me2: H4 arginine 3 dimethylation
HCC: Hepatocellular carcinoma
HD: Huntington’s disease
HDAC1: Histone deacetylase 1
HER2: Human epidermal growth factor receptor 2
HIF-1α: Hypoxia-inducible factor-1 alpha
HIV-1: Human immunodeficiency virus type 1
HMGCR: 3-Hydroxy-3-methylglutaryl-CoA reductase
HNRNPF: Heterogeneous nuclear ribonucleoprotein F
HOXA1: Homeobox A1
HOXA3: Homeobox A3
HOXA5: Homeobox A5
HOXA9: Homeobox A9
HPK1: Hematopoietic progenitor kinase 1
HSP90: Heat shock protein 90
HSV1: Herpes simplex virus 1
HUWE1: HECT domain‐containing E3 ubiquitin ligase 1
IL-17F: Interleukin-17F
IGF1R: Insulin-like growth factor 1 receptor
IGFBP4/5: Insulin-like growth factor binding protein 4/5
IKZF1: IKAROS family zinc finger 1
IRF4: Interferon regulatory factor 4
ITGA6: Integrin subunit alpha 6
JMJD: Jumonji C domain-containing
JmjC: Jumonji C
KDMs: Lysine demethylases
KIF14: Kinesin family member 14
KSHV: Kaposi’s sarcoma-associated herpesvirus
LMO2: LIM domain only 2
LSCs: Leukemia stem cells
LSD1: Lysine-specific demethylase 1
MALAT1: Metastasis-associated lung adenocarcinoma transcript 1
MCL-1: Myeloid cell leukemia 1
MDSC: Myeloid-derived suppressor cells
MIF: Migration inhibitory factor
MLL2: Mixed-lineage Leukemia 2
NAD^+^: Nicotine adenine dinucleotide
NAFLD: Non-alcoholic fatty liver disease
NASH: Non-alcoholic steatohepatitis
NF-κB: Nuclear factor kappa-B
NOXA: Phorbol-12-myristate-13-acetate-induced protein 1
NR4A1: Nuclear receptor subfamily 4 group A member 1
NUP98: Nucleoporin 98
PAF1: Polymerase-associated factor 1
PARP: Poly(ADP-ribose) polymerase
PARP16: Poly(ADP-ribose) polymerase 16
PCa: Prostate cancer
PCNA: Proliferating cell nuclear antigen
PCSK9: Proprotein convertase subtilisin/kexin type 9
PD: Parkinson’s disease
PHB1: Prohibitin 1
PHGDH: Phosphoglycerate dehydrogenase
PI3K: Phosphoinositide-3-kinase
PKA: Protein kinase A
PKM2: Pyruvate kinase M2
PLGA: Poly lactic-co-glycolic acid
PPARγ: Peroxisome proliferator-activated receptor gamma
PRC1: Polycomb repressive complex 1
PRC2: Polycomb repressive complex 2
PRMT1: Protein arginine methyltransferase 1
PROTAC: Proteolysis-targeting chimera
PTMs: Post-translational modifications
PUMA: P53 upregulated modulator of apoptosis
RCCD1: RCC1 domain containing 1
REST: RE-1 silencing transcription factor
RFX5: Regulatory factor X 5
RIG-I: Retinoic acid-inducible gene I
ROCK1: Rho-associated coiled-coil containing protein kinase 1
ROCK2: Rho-associated coiled-coil containing protein kinase 2
Rpl27a: Ribosomal protein L27a
RPS6: Ribosomal protein S6
RUNX3: Runt-related transcription factor 3
SASP: Senescence-associated secretory phenotype
SAT1: Spermidine/spermine N1-acetyltransferase 1
SERMs: Selective estrogen receptor modulators
SLE: Systemic lupus erythematosus
SNCA: Synuclein alpha
SOX9: SRY-box transcription factor 9
SPARCL1: Secreted protein acidic and rich in cysteine like protein 1
SPI1: *Salmonella* Pathogenicity island I
SUMOylation: Small ubiquitin-like modifier conjugation
T-ALL: T-cell acute lymphoblastic leukemia
TBX15: T-box transcription factor 15
TCL: TC10-like
TEAD1: TEA domain transcription factor 1
TGF-β: Transforming growth factor-beta
TIAM1: T-cell lymphoma invasion and metastasis 1
TLR4: Toll-like receptor 4
TME: Tumor microenvironment
TMZ: Temozolomide
TNBC: Triple-negative breast cancer
TNC: Tenascin C
TNFAIP3: Tumor necrosis factor alpha-induced protein 3
TPR: Tetratricopeptide repeat
TRAF6: TNF receptor-associated factor 6
U2AF65: U2 small nuclear ribonucleoprotein auxiliary factor 65-kilodalton subunit
UPR: Unfolded protein response
VDR: Vitamin D receptor
VEGF: Vascular endothelial growth factor
WWP1: WW domain-containing E3 ubiquitin protein ligase 1
XLMR: X chromosome-linked mental retardation
XRCC1: X-ray repair cross complementing protein 1
YTHDF3: YTH N6-methyladenosine RNA binding protein 3
YY1: Yin Yang 1
2-OG: 2-Oxoglutarate
